# Enhanced metabolic detoxification is associated with fluroxypyr resistance in *Bassia scoparia*

**DOI:** 10.1002/pld3.560

**Published:** 2024-01-24

**Authors:** Olivia E. Todd, Eric L. Patterson, Eric P. Westra, Scott J. Nissen, André Lucas Simões Araujo, William B. Kramer, Franck E. Dayan, Todd A. Gaines

**Affiliations:** ^1^ United States Department of Agriculture – Agriculture Research Service (USDA‐ARS) Fort Collins Colorado USA; ^2^ Department of Agricultural Biology Colorado State University Fort Collins Colorado USA; ^3^ Department of Plant, Soil, and Microbial Sciences Michigan State University East Lansing Michigan USA; ^4^ Department of Plants, Soils & Climate Utah State University Logan Utah USA

**Keywords:** herbicide resistance, NTSR, synthetic auxin

## Abstract

Auxin‐mimic herbicides chemically mimic the phytohormone indole‐3‐acetic‐acid (IAA). Within the auxin‐mimic herbicide class, the herbicide fluroxypyr has been extensively used to control kochia ( *Bassia scoparia* ). A 2014 field survey for herbicide resistance in kochia populations across Colorado identified a putative fluroxypyr‐resistant (Flur‐R) population that was assessed for response to fluroxypyr and dicamba (auxin‐mimics), atrazine (photosystem II inhibitor), glyphosate (EPSPS inhibitor), and chlorsulfuron (acetolactate synthase inhibitor). This population was resistant to fluroxypyr and chlorsulfuron but sensitive to glyphosate, atrazine, and dicamba. Subsequent dose‐response studies determined that Flur‐R was 40 times more resistant to fluroxypyr than a susceptible population (J01‐S) collected from the same field survey (LD_50_ 720 and 20 g ae ha^−1^, respectively). Auxin‐responsive gene expression increased following fluroxypyr treatment in Flur‐R, J01‐S, and in a dicamba‐resistant, fluroxypyr‐susceptible line 9,425 in an RNA‐sequencing experiment. In Flur‐R, several transcripts with molecular functions for conjugation and transport were constitutively higher expressed, such as glutathione S‐transferases (GSTs), UDP‐glucosyl transferase (GT), and ATP binding cassette transporters (ABC transporters). After analyzing metabolic profiles over time, both Flur‐R and J01‐S rapidly converted [^14^C]‐fluroxypyr ester, the herbicide formulation applied to plants, to [^14^C]‐fluroxypyr acid, the biologically active form of the herbicide, and three unknown metabolites. The formation and flux of these metabolites were faster in Flur‐R than J01‐S, reducing the concentration of phytotoxic fluroxypyr acid. One unique metabolite was present in Flur‐R that was not present in the J01‐S metabolic profile. Gene sequence variant analysis specifically for auxin receptor and signaling proteins revealed the absence of non‐synonymous mutations affecting auxin signaling and binding in candidate auxin target site genes, further supporting our hypothesis that non‐target site metabolic degradation is contributing to fluroxypyr resistance in Flur‐R.

## Significance Statement

Herbicide resistance is an ever‐present issue in weeds of cropping and rangeland systems. Every characterized herbicide resistance mechanism contributes to a working database used to address herbicide resistance in an agricultural or open‐space setting. Knowing the exact resistance mechanism helps public sector and industry researchers understand why herbicide applications are failing, and if resistant plants can still be controlled with other herbicide modes of action. In the kochia line Flur‐R, there is strong evidence to support a non‐target site resistance mechanism, specifically herbicide degradation via increased enzymatic activity. Increased fluroxypyr degradation represents a novel resistance mechanism to fluroxypyr in *Bassia scoparia*.

## 1 INTRODUCTION

**FIGURE 1:**
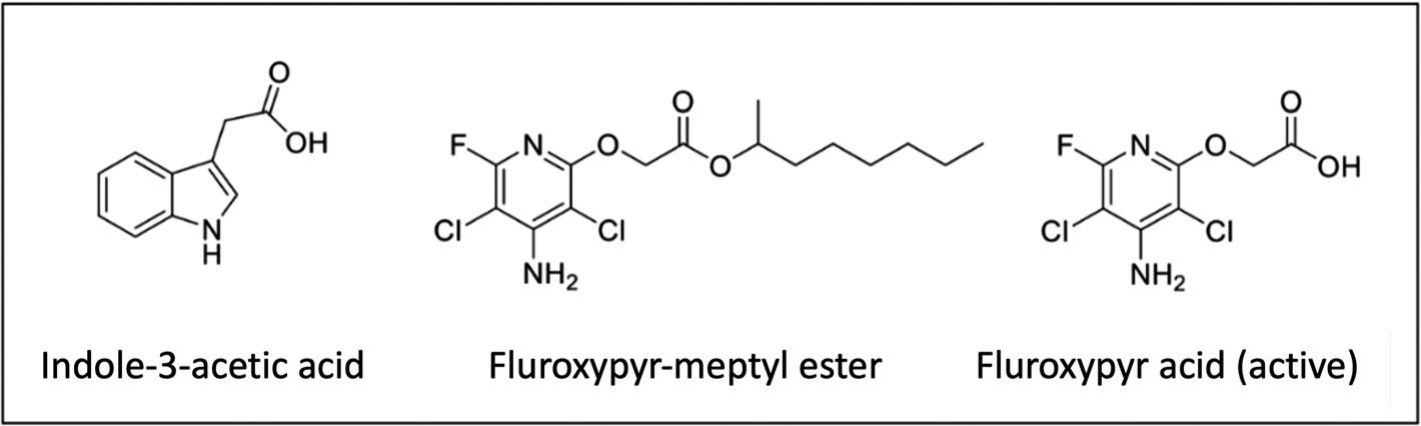
Chemical structure of indole‐3‐acetic acid (IAA), fluroxypyr‐meptyl ester (fluroxypyr‐ester) included in the formulated commercial products, and fluroxypyr‐acid, the biologically active form of the herbicide. Deesterification of fluroxypyr‐meptyl ester frees the carboxyl group shown in fluroxypyr‐acid, which plays a key role in plant perception related to the auxin signaling pathway in relation to the ring structure found throughout auxin‐mimic herbicide chemistry.

Kochia (*Bassia scoparia*) is an invasive, annual, broadleaf tumbleweed that is problematic in agronomic settings, open spaces, and rangeland in the U.S., specifically across the Great Plains. Herbicides are the most prescribed method for kochia control in the U.S. Herbicide resistance in kochia is widespread, with resistance reported to multiple modes of action including ALS inhibitors (Group 2), glyphosate (Group 9), auxin‐mimics (Group 4), and atrazine (Group 5), as well as cross‐resistance to more than one Group 4 auxin‐mimic herbicide (Geddes, Owen, et al., 2021; Kumar, Currie, et al., 2019; Kumar, Jha, et al., 2019). Furthermore, multiple resistance is increasingly common, where one population is simultaneously resistant to more than one mode of action (Varanasi et al., 2015). The prevalence of glyphosate, atrazine, and ALS inhibitor‐resistant kochia has resulted in increased use of auxin‐mimic herbicides for kochia management, mainly dicamba and fluroxypyr, in no‐till fallow, wheat, and corn in the Great Plains region (Kumar, Jha, et al., 2019). While auxin‐mimics have been used for more than 70 years, resistance evolution to this mode of action has lagged behind other herbicide modes of action (Busi et al., 2018). Despite this lag, recent evidence suggests that auxin‐mimic resistance and multiple resistance in kochia is increasing (Geddes et al., 2022). Nine reports of auxin‐mimic resistance across six U.S. states and two Canadian provinces have described resistance in kochia populations as either resistant to dicamba alone or resistant to both dicamba and fluroxypyr (Geddes, Ostendorf, et al., 2021; Heap, 2023; Jha et al., 2015; Kumar, Currie, et al., 2019). These herbicides mimic the phytohormone indole‐3‐acetic‐acid (IAA) because they are chemically similar and induce auxin response gene transcription following application in weeds (Grossmann, 2010; McCauley et al., 2020; Pettinga et al., 2018; Xu et al., 2022) (Figure 1).

IAA is an auxin plant growth hormone that is responsible for gravitropism and response to light stimuli; however, it impacts several other growth phenotypes as well (Zhao, 2010). While auxins are involved in many cellular processes and signaling with other phytohormones, their function can be understood at the cellular level to primarily coordinate cell elongation (Perrot‐Rechenmann, 2010). In plants, auxin homeostasis is tightly regulated through a suite of biosynthesis pathways, cellular transport, feedback inhibition, oxidation, and conjugation (Rosquete et al., 2012). When IAA reaches high levels in the plant, polar auxin carriers such as Pin‐formed (PIN) efflux transporters, ATP binding cassettes (ABC class B) and Auxin resistant‐1/like AUX1s influx carriers (AUX/LAX) help maintain IAA homeostasis and gradients (Cho & Cho, 2013). Because the auxin‐mimic herbicide fluroxypyr is chemically similar to and behaves like IAA, it is hypothesized that PIN, ABCs, and AUX/LAX are able to direct the flow of fluroxypyr throughout the plant. In addition, fluroxypyr is a weak acid that can translocate in the plant based on its pKa and log Kow. Fluroxypyr also binds to Auxin signaling F‐box 5, a member of the Transport inhibitor response1/Auxin signaling F‐box (TIR1/AFB) receptor family (Lee et al., 2014). When applied, fluroxypyr and IAA act to stabilize the complex formed between AFB5 and the auxin‐dependent transcriptional regulator Indole‐3‐acetic acid inducible (Aux/IAA) proteins. Upon creation of this coreceptor‐ligand complex, Aux/IAA proteins are ubiquitinated, degraded, and no longer negatively regulate Auxin Response Factors (ARF). These ARF proteins are seated on the Auxin Response Element (AuxRE) in auxin‐mediated gene promotors (Teale et al., 2006).

In Arabidopsis, plants treated with IAA or auxin‐mimic herbicide (2,4‐D) showed expression of early response genes such as Aux/IAAs and small auxin‐up RNAs (SAURs). Other auxin‐induced genes included 1‐aminocyclopropane‐1 synthase (ACS), the first committed step in ethylene production, and GH3, an auxin homeostasis gene. These genes were transcribed within minutes of high auxin perception (Guilfoyle, 1999; Paponov et al., 2008; Raghavan et al., 2005). Many other phytohormone responses are also regulated by auxin perception, such as Cytokinin oxidase (CXK6), brassinosteroid biosynthesis gene BAS1, and several gibberellin‐related genes. Regulation of multiple hormone‐related genes suggests that the relationship between phytohormones and auxin response is complex (Paponov et al., 2008). When the auxin‐mimic herbicide fluroxypyr is applied to a plant, the resulting phenotypic response is changes in leaf angle, stem‐twisting, stem‐thickening, and lack of new growth at the meristem.

Herbicide resistance is categorized as either a target site or non‐target site (Gaines et al., 2020). Target site resistance is defined as a change (either in conformation or in expression) in a herbicide target protein. These changes often decrease herbicide binding affinity or in the case of overexpression, make it so the entire target protein pool is unable to be entirely inhibited (Murphy & Tranel, 2019). LeClere et al. (2018) reported resistance to the auxin‐mimic herbicide dicamba in kochia was due to the target‐site mutation Gly127Asn in *IAA16*, which affects the formation of the coreceptor‐ligand complex. More recently, Figueiredo, Küpper, et al. (2022) characterized a 27‐nucleotide deletion in the degron tail region of the gene encoding Aux/IAA2 that confers 2,4‐D and dicamba resistance in Indian hedge mustard (*Sisymbrium orientale*). This deletion also affects the formation and stability of the co‐receptor‐ligand complex. Non‐target site resistance is broadly recognized as all other methods unrelated to target site resistance and is often exemplified by metabolic detoxification of an herbicide, herbicide sequestration, or a variant in a metabolism‐catalyzing enzyme which may have a downstream effect by reducing the efficacy of the herbicide (Delye, 2013). Reduced translocation of 2,4‐D via auxin transport proteins was reported by Goggin et al. (2019) in wild radish (*Raphanus raphanistrum*). A 2,4‐D resistant population of waterhemp (*Amaranthus tuberculatus*) rapidly metabolized 2,4‐D via CYP450 5‐OH hydroxylation and subsequent amino acid and sugar conjugation reactions, which produced less phytotoxic metabolites that lost auxin signaling activity (Figueiredo et al., 2018; Figueiredo, Barnes, et al., 2022). With both target site and non‐target site resistance mechanisms described for auxin‐mimic resistance (Todd et al., 2020), both possibilities are investigated in this study.

Our research objectives were to (1) **distinguish the application rate at which the fluroxypyr‐resistant kochia line (Flur‐R) is resistant using a herbicide dose‐response,** (2) **determine whether fluroxypyr‐resistant kochia has any differences in absorption, translocation, or metabolism,** and (3) **identify potential candidate genes that may contribute to fluroxypyr resistance in Flur‐R using RNA sequencing and alignment to the kochia genome assembly** (Hall et al., 2023).

In our work, Flur‐R converted fluroxypyr‐ester into fluroxypyr‐acid and subsequent metabolites at a faster rate than a susceptible line. Furthermore, the resistant line produced a metabolite that was not detected in the susceptible line. The results from an RNA‐seq fluroxypyr‐induced differential expression analysis showed increased transcript expression of cellular transporters, cytochrome P450 monooxygenases (CYP450), glutathione s‐transferases (GSTs), and UDP‐glucosyl transferase (GTs) in the resistant plants. Taken together, these data suggest that metabolic detoxification of fluroxypyr may be the mechanism of fluroxypyr resistance in Flur‐R.

## 2 MATERIALS AND METHODS

### 2.1 Plant materials

In 2014, 171 kochia populations were collected from a field survey conducted in eastern Colorado (Westra et al., 2019). These populations were screened at a single dose of dicamba, fluroxypyr, and glyphosate to test for resistance. One population, Flur‐R, was found to have a few individuals (<2%) surviving a single fluroxypyr dose of 157 g ae ha^−1^ (label rate for use in wheat) (Starane Ultra, Dow Agrosciences, Indianapolis, IN). Survivors at 157 g ae ha^−1^ were selected and allowed to bulk pollinate. After two more generations of selection at both 157 g ae ha^−1^ and 314 g ae ha^−1^, the surviving individuals were cross‐pollinated. The progeny of these plants were found to be uniformly resistant to 314 g ae ha^−1^ fluroxypyr. During the bulking stages, groups of three to four plants were planted in one gallon pots and covered with pollination bags (Vilutis & Co., Frankfort, IL) to allow for cross‐pollination. Seed was harvested per pot, hand threshed, and cleaned using an air‐column blower. Seeds were stored at 4 C and planted in the spring in a greenhouse maintained at 25 C with a 16 h photoperiod supplemented with metal‐halide lamps (400 μmol m^−2^ s^−1^). An inbred dicamba‐resistant/fluroxypyr susceptible population (9425) homozygous for a G127N mutation in the *IAA16* gene (LeClere et al., 2018; Preston et al., 2009) and a fluroxypyr susceptible field population from the 2014 eastern Colorado field study (J01‐S) (Westra et al., 2019) were included in the dose‐response and single dose screening as susceptible controls.

### 2.2 Fluroxypyr and Dicamba dose response

Seeds of Flur‐R, J01‐S, and 9,425 were planted in 1.5 cm^2^ 280‐count plug flats (American Clay Works Supply, Denver, CO). Plants were sub‐irrigated and thinned down to one plant per cell. When plants were approximately 4–5 cm tall, uniform seedlings were transplanted to 4 cm^2^ plastic pots containing SunGro potting mix (American Clay Works Supply, Denver, CO). Plants were kept in the greenhouse under the conditions previously described. Plants were sub‐irrigated once a week for three weeks until the plants reached 10 cm in height. A randomized complete block design was used for each dose, with one plant per pot, four plants per dose, and three replications for a total of 12 treated plants. The dose‐response for fluroxypyr included the following eight rates: 0, 20, 40, 80, 157, 314, 628, and 1,256 g ae ha^−1^ fluroxypyr (Starane Ultra, Corteva Agrisciences, Indianapolis, IN). The dicamba doses included 0, 35, 70, 140, 280 (1x), 560, 1,120, and 2,240 g ae ha^−1^ (Engenia, BASF, Research Triangle Park, NC) mixed with Induce (NIS, .25% v/v, Helena Agri‐Enterprises, LLC, 24330 US‐34 Greely, CO 80631). Applications were made with a DeVries Generation 4 Research Track Sprayer (DeVries Manufacturing, Hollandale, MN, 86956) equipped with a TeeJet (TeeJet Technologies, 1801 Business Park drive, Springfield, IL) 8002EVS nozzle calibrated to deliver 187 L ha^−1^. Plant height was measured by recording the distance in centimeters from the soil surface to the newest leaf in the apical meristem before treating and was measured again 30 days after treatment. Survival (dead or alive) was also recorded 30 days after treatment. An individual was considered “dead” if it displayed severe epinasty, stem thickening, and yellowing, and had no new growth at the axillary or primary meristems after 30 days. An individual was considered “alive” if it displayed minimal to no epinasty or stem thickening, had no yellowing, and had new growth at the axillary or primary meristems after 30 days. All individuals were uniform for either dead or alive phenotypes, with no intermediate responses. Percent survival was chosen for fluroxypyr resistance assessment because while percent change in height can accurately differentiate between resistant and susceptible plants, for this population it did not accurately represent an actively growing plant in the individuals where axillary meristem growth was the primary source of regrowth.

For data analysis, the response variable “Percent Survival” was created by transforming binary data according to the equation:

(1) $$Y=\left(\frac{N_{1}}{N_{\text{total}}}\right)*100$$

Where *Y* is the percent survival at each calculated dose, *N*_*1*_ is the number of individuals marked as “alive” according to the parameters above. *N*_*total*_ is the number of individuals per rate.

The response variable “Percent Change in Height” over 30 days was normalized using the following equation:

(2) $$Y=\left(X_{∆\text{Height}}/A_{\mathrm{Avg}}\right)*100$$

Where *Y* is the change in height as a percent of the 0 g ae ha^−1^ rate for each population. $X_{∆\text{Height}}$ is the change in height in centimeters for an individual from day 0 to 30 days, and *A*_*Avg*_ is the average change in height for individuals at the 0 g ae ha^−1^ rate for the population being measured. The model used by the drm package in R did not converge for the J01‐S or 9,425 lines using “Percent Change in Height (% control)” as a response variable due to the non‐sigmoidal behavior of the curve, so “Percent Survival” data were analyzed using a three‐parameter log‐logistic model (Knezevic et al., 2007), which was the best model by a lack‐of‐fit test (“ModelFit” command) from the drc package in R (R Core Team, 2020) with the equation:

(3) $$Y=\frac{d}{1+\exp\;\left[b\left(\log\;\left[\left(x-\log\right)\;\left(e\right)\right]\right)\right]\;}$$

where *Y* is the percent survival 30 days after treatment, *d* is the upper limit parameter, *b* is the regression slope, *x* is the dose of either fluroxypyr or dicamba in g ae ha^−1^ and *e* is the dose at which 50% mortality is achieved (Table 1). The homoscedasticity and normality of the data were assessed and met model assumptions. The data were averaged per treatment and the standard error of the mean is presented per dose. “Rate” and “Population” were used as predictor variables and the experiment was repeated. Homogeneity of variance test was conducted to determine if the repeated dose‐response data could be combined for analysis. The data could not be combined, so the repeated experiment was analyzed separately and consistent results were identified.

**TABLE 1:** Parameters for fluroxypyr dose–response data in kochia ( *Bassia scoparia* ) populations Flur‐R, 9425, and J01‐S. Parameters of the fluroxypyr and dicamba dose‐responses for percent survival parameters are described in Equation 3 for Flur‐R, fluroxypyr sensitive line J01‐S, and fluroxypyr sensitive/dicamba‐resistant line 9,425. Flur‐R shows a significant resistance factor ratio (R/S) of 36 and 40 relative to 9,425 and J01, respectively. (*b,d*) Lower and upper limits of regression parameters, respectively. (*LD*_*50*_) the dose (g ae ha^−1^) of fluroxypyr where 50% mortality occurs for each population. (*R/S)* The ratio of resistant LD_50_ to either susceptible LD_50_ and associated *p*‐values.

Herbicide										
	Fluroxypyr					Dicamba				
*Line*	*b* ( ±SE)	*d* ( ±SE)	LD_50_ ( ±SE)	R/S	*p*‐value	*b* ( ±SE)	*d* ( ± SE)	LD_50_ ( ±SE)	R/S	*p*‐value
	‐‐‐‐‐g ae ha^−1^‐‐‐‐‐					‐‐‐‐‐g ae ha^−1^‐‐‐‐‐				
Flur‐R	7.3 (8.4)	94.5 (3.6)	720 (110.3)	36–40	<.001	7.4 (5.2)	100.0 (4.9)	56 (8.5)	‐‐	‐‐
9,425	8.5 (37.7)	100.0 (8.8)	20 (1.5)	‐‐	‐‐	85.1 (10.0)	91.7 (2.72)	415 (10.0)	6–7	<.001
J01‐S	3.1 (1.8)	100.0 (8.8)	18 (2.7)	‐‐	‐‐	9.3 (8.5)	100.0 (4.93)	64 (5.4)	‐‐	‐‐

### 2.3 Glyphosate, atrazine, and Chlorsulfuron single dose screening

Flur‐R and J01‐S seeds were planted in 4 cm^2^ plastic pots containing SunGro potting mix. Plants were sub‐irrigated and thinned down to one plant per pot and kept at greenhouse conditions previously described. When plants were approximately 7 cm in height, plants were treated with one of the following herbicides (*n* = 72 plants per herbicide): atrazine (Aatrex 4 L, Syngenta, Greensboro, NC, 2240 g ai ha^−1^, 1% crop oil concentrate), chlorsulfuron (Telar XP, Bayer CropScience, St. Louis, MO, 137 g ai ha^−1^), or glyphosate (RoundUp Powermax, Monsanto Company, St. Louis, MO, 870 g ae ha^−1^, 2% w/v ammonium sulfate). All treatments were applied with the same equipment and nozzle type described above. Survival (dead or alive) was assessed 30 days after treatment. In a post hoc analysis, a random number generator was used to assign each of the 72 individuals to one of three blocks with *n* = 24 to serve as replicates. The standard error of the mean was calculated using the standard deviation from this analysis.

### 2.4 Kompetitive allele‐specific PCR (KASP)

Approximately 200 mg of meristem tissue was harvested from 20 individuals each of Flur‐R and 5 individuals for the mutant and wild type checks. Flur‐R individuals were verified as resistant by spraying with 157 g ae ha^−1^ fluroxypyr. Tissue was put into a 1.5 ml Eppendorf tube and flash frozen in liquid nitrogen. DNA extraction protocol was adapted from Aboul‐Maaty and Oraby (2019) using the established CTAB method. DNA purification was checked using a NanoDrop2000 and diluted to 5 ng uL^−1^. The FAM fluorophore (in bold) was added to the forward primer specific to the G127N *IAA16* double mutation (allele‐specific sequence in italics) endowing a protein change from wildtype GWPPV to NWPPV in kochia described by LeClere et al. (2018) (5’**GAAGGTGACCAAGTTCATGCT**TGTTCTTCAGGACACAAGTTGTA*AA*) and the HEX fluorophore (in bold) was added to the forward primer specific to the wild type sequence (in italics) (5’**GAAGGTCGGAGTCAACGGATT**TGTTCTTCAGGACACAAGTTGTA*GG*). One universal reverse primer (5’AGTTTGATCATCGGACGTCTTCTT) and the forward primers were designed with IDT PrimerQuest. The KASP protocol and specific mix ratios are published on protocols.io at dx.doi.org/10.17504/protocols.io.dm6gpj9njgzp/v1. Fluorescence was recorded at the end of every cycle. Fluorescence at the 35th cycle was used for the allelic discrimination data. Data were plotted using GraphPad Prism version 8.4.2. Genotypes were assigned manually as homozygous wildtype, homozygous mutant, or heterozygous.

### 2.5 Plant material for Fluroxypyr absorption, translocation, and metabolism

Seeds from Flur‐R and J01‐S were sown into plug flats filled with SunGro potting mix and grown on a light shelf under 700 μmol m^−2^ s^−1^ of light at 25 C. When the plants reached 3–4 cm tall, 50 seedlings from each line were washed of soil in the roots and transplanted into a 25 ml Eppendorf tube filled with silica sand and fertilized with three granules of Osmocote. Uniform plants that were 4–5 cm tall and had recovered from transplanting were used in all subsequent absorption, translocation, and metabolism experiments.

### 2.6 Fluroxypyr absorption and translocation

Flur‐R (*n* = 24) and J01‐S (*n* = 24) plants were sprayed with 157 g ae ha^−1^ fluroxypyr using a track sprayer as described in section 2.2. The third and fourth youngest leaves in the meristem were protected from the broadcast application using aluminum foil. Immediately after applying fluroxypyr, the two covered leaves were treated with five 1 μl drops of the spray solution spiked with 3.1 kBq [^14^C]‐fluroxypyr. Absorption and translocation were monitored over a 196 h time course, with time points at 6, 12, 24, 48, 96, and 192 hours after treatment (HAT). Four Flur‐R and four J01‐S were harvested at each timepoint. Treated leaves were removed and washed in 5 ml 90% water, 10% methanol, and .5% non‐ionic surfactant. The leaf wash was mixed with 10 ml scintillation cocktail (Ecoscint XR) and radioactivity was measured using a liquid scintillation counter (LSC) (TRI‐CARB 2300TR, Packard Instruments Co., USA). Roots were washed with 5 ml water. Root wash and the silica sand rinse solution were vortexed for 3 seconds, and 1 ml of the root wash and sand rinse mixture was added to 10 ml scintillation cocktail to measure root exudation. Plants were sectioned and separated as follows: above‐treated leaves, treated leaves, below‐treated leaves, and root biomass. Each separate plant part was dried and oxidized using a biological oxidizer (Model OX500, R. J. Harvey Instrument Co., USA). The released ^14^C‐CO_2_ was collected by a ^14^C trapping cocktail (OX161, R.J. Harvey Instrument Co., USA). Radioactivity was quantified by LSC. One individual per timepoint was left intact, dried, and used for phosphor imaging (Typhoon Trio, GE Healthcare). Dried plants were separated and oxidized in the same manner as described above after imaging.

The experiment was repeated. Percent absorption and translocation were calculated as follows from Figueiredo et al. (2018) and maximum percent absorption was determined using a method described by Kniss et al. (2011) in R.

%Habs=C14ot/C14ot+C14wl×100%Htr=100−C14al/C14al+C14ot×100

Where “%*H*_abs_” is percent absorption of [^14^C]‐fluroxypyr ester, “^14^C ot” is the sum DPM from the oxidation of all plant parts, and “^14^C ot + ^14^C wl” is the sum DPM from the oxidation of all plant parts and counts washed from the treated leaf. For herbicide translocation studies, “%*H*_tr_” is percent translocation of [^14^C]‐fluroxypyr ester out of the treated leaf through the rest of the plant, “^14^C al” is the DPM [^14^C]‐fluroxypyr ester counted in the treated leaf.

### 2.7 Fluroxypyr metabolism

Fluroxypyr metabolism was evaluated by treating Flur‐R and J01‐S plants as previously described. Plants were sprayed with fluroxypyr while two leaves were protected from the spray solution. Those protected leaves were then treated with five 1 μl drops of the spray solution spiked with 25 kBq [^14^C]‐fluroxypyr. The time course was the same as previously described with four repetitions per timepoint. Treated leaves were removed and washed as previously described. The washed treated leaves were placed back with the remaining whole‐plant tissue and flash‐frozen in liquid nitrogen. Whole plants were then finely ground in a glass test tube with liquid nitrogen and a glass rod. Five milliliter extraction solution (90% water, 9% acetonitrile, 1% acetic acid) was added to each tube and samples were shaken for 30 min. The extraction solution was transferred to a .45 μm filter tube which was rinsed with an additional 5 ml extraction buffer and centrifuged at ~2,600 × g for 10 min to separate liquid from ground plant material. The extraction buffer that passed through the .45 μm filter was transferred to C‐18 cartridges preconditioned with 1 ml 100% acetonitrile (Waters Co., Sep‐Pak Plus). Using a vacuum manifold, the extraction buffer was pulled through the solid phase extraction cartridge. Bond solutes were eluted from the C18 cartridge with 5 ml 100% acetonitrile and samples were then evaporated to dryness in a fume hood. Solvent A (500 μL) consisting of 10% acetonitrile and 1% formic acid was added to each tube. Each sample was filtered through 25 μm nylon filters (Nalgene) into an injection vial with a 500 μL insert. High‐Pressure Liquid Chromatography (HPLC) (Hitachi Instruments, Inc.,) was used to separate radiolabelled fluroxypyr‐ester, fluroxypyr acid, and metabolites. The HPLC was equipped with a C‐18 column (4.6 mm by 150 mm column, Zorbax Eclipse XDB‐C18, Agilent Technologies) and inline radio‐detector (FlowStar LB 513, Berthold Technologies GmbH & Co.) with a YG‐150‐U5D solid scintillation flow cell (150 μl). The injection volume was 200 μL. Radiolabelled fluroxypyr‐ester had a retention time of 9.8 min, while fluroxypyr acid had a retention time of 2.8 minutes (protocol published on protocols.io at dx.doi.org/10.17504/protocols.io.kqdg39yopg25/v1).

### 2.8 Plant material and treatment for RNA sequencing

Seedlings from Flur‐R, 9425, and J01‐S were grown as described above. When the plants reached 7–10 cm tall, 20 of the most uniform seedlings from each line were treated as follows: All plants were sprayed with water and .01 g meristem tissue was harvested for the untreated RNA‐sequencing timepoint. Tissue was flash‐frozen in liquid nitrogen. The same 20 plants per line were treated 24 h later with 157 g ae ha^−1^ fluroxypyr, the labeled dose to control kochia in wheat. Approximately .01 g of meristem tissue was harvested at 3 and 10 h after fluroxypyr treatment for the remaining two RNA‐sequencing timepoints. Herbicide and water applications were made with a track sprayer as described in section 2.2. All plants were in the vegetative stage, except for one Flur‐R individual and three J01‐S individuals, which were in the early flowering stage at the time of tissue harvest. After 30 days, resistance response was measured and four individuals per timepoint per line were selected for RNA‐sequencing.

### 2.9 RNA extraction, sequencing and quantification

The RNA‐sequencing experiment was conducted first by extracting total RNA following the protocols in the QIAGEN RNeasy plant mini kit in six batches containing two individuals of each line to minimize batch effects. The kit was used to extract RNA from the top three fully expanded apical meristem leaves of Flur‐R, 9425, and J01‐S of 5–7 cm tall kochia at 0, 3, and 10 h after 157 g ai ha^−1^ fluroxypyr treatment. The final elution volume was 30 μl. Total RNA samples were diluted to a range of 500–10,000 pg μL^−1^ for quality check using an Agilent ScreenTape. Samples that had an RIN score above 6 were submitted to BGI Technologies for quality check following their sample submission guidelines. Following the quality check by BGI, 30 samples were used for sequencing. From the total RNA, mRNA enrichment was performed by rRNA depletion. Reverse transcription of the mRNA was performed with random N6 primers followed by end repair and A‐tail and adapter ligation to the fragments. After PCR amplification, single‐strand separation and single‐strand circularization were conducted to sequence paired‐end 100 base pair fragments with the BGISEQ sequencing platform. In total, 2.8 billion reads were produced, resulting in 92–97 million 100 bp reads per sample.

### 2.10 Differential expression and variant analysis

To analyze the transcriptome of Flur‐R, we sequenced RNA from 4 plants each of fluroxypyr resistant Flur‐R, and two fluroxypyr susceptible lines J01‐S and 9,425. BGI Seq was used to obtain between 91 and 95 million clean reads per sample (BGI Bioinformatics, San Jose, CA). Individual fasta files were uploaded to the remote research computing resource SUMMIT (Jonathon Anderson et al., 2017) and files were quality‐checked with FastQC (version .11.9). Adapters were trimmed by BGI Bioinformatics company after sequencing and quality check. Q20 scores were between 96% and 98%. Alignment was made to the coding sequence of the *B. scoparia* genome assembly version 2 (Hall et al., 2023) using HISAT2 (version 2.2.0 [Kim et al., 2019]), and alignment ranged between 59% and 63% for all individuals. Percent unmapped reads ranged between 46% and 51%, and percent uniquely mapped genes ranged from 43% to 48%. Approximately 4% of reads were multi‐mapped (Table S1). Reads were assigned to features using featureCounts in the Subread package (version 2.1, Liao et al., 2014). Differential expression was conducted with resultant reads for each gene feature using the DESeq2 package (version 1.28.1) in the statistical software R (version 4.2 (R Core Team, 2020)). Reads were transformed to logarithmic fold change log2 and compared across biological replicates for each population. For each population, the untreated condition was compared to either the 3 or 10 h timepoint to determine expression. Mean normalized counts per gene, an adjusted pvalue of < .05, and log2 fold change > .5 were the pre‐filtering parameters used by DESeq2 for optimal significant genes below the false discovery rate (FDR) of <.05.

Sorted and indexed bam files were run through the variant calling software Platypus (version .8.1 [Rimmer et al., 2014]) to detect single and mono‐nucleotide polymorphisms, short and long indels, as well as chromosome rearrangement. The output file was used with the software SnpEff (version 4.3 [Cingolani et al., 2012]) to annotate the variants called from Platypus and to provide effect predictions. Specific genes annotated with involvement in the auxin signaling pathway or metabolic herbicide resistance were targeted for variant analysis by checking chosen genes against a merged variant file for all individuals of each population. Presence or absence of variants was validated with Integrative Genomics Viewer (IGV) (Robinson et al., 2017).

## 3 RESULTS

### 3.1 Fluroxypyr and Dicamba dose response

**FIGURE 2:**
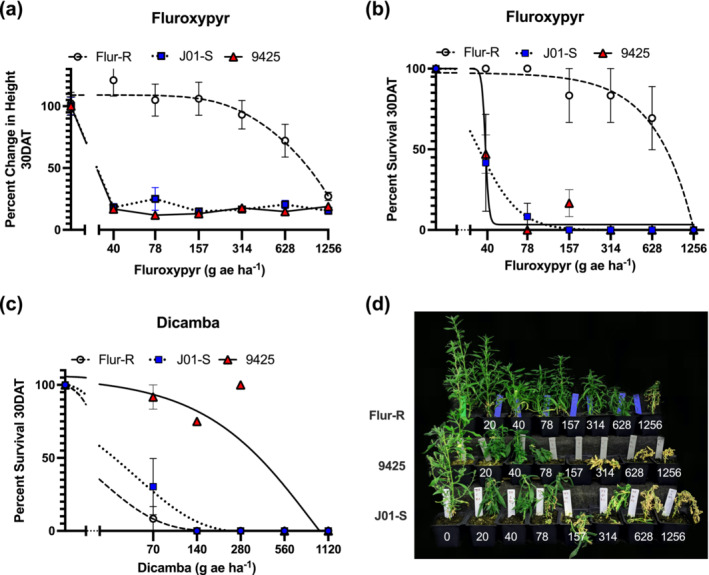
Dose‐response data for (a,b) fluroxypyr (no adjuvant) and (c) dicamba (+ .25% NIS) demonstrated fluroxypyr resistance and dicamba sensitivity in fluroxypyr resistant line Flur‐R. X‐axis is represented in a log10 scale. (a) Percent change in height as a percent of the untreated control 30 days after treatment with fluroxypyr showed a 25% reduction in height in Flur‐R at 628 g ae ha^−1^ (four times the label rate of 157 g ae ha^−1^). (b) Percent survival for Flur‐R with greater than 70% survival to fluroxypyr at 628 g ae ha^−1^ (LD_50_ = 720, *p* = <.001). The population 9,425 was susceptible to fluroxypyr (LD_50_ = 20 g ae ha^−1^, *p* < .001). (c) Flur‐R was susceptible to dicamba (LD_50_ = 56 g ae ha^−1^, *p* < .001) and the known dicamba‐resistant line, 9,425, was resistant to dicamba up to 280 g ae ha^−1^. Error bars represent SEM. (d) Singular plants represent the average line response at each dose of fluroxypyr where 157 g ae ha^−1^ represents the label rate.

Flur‐R was confirmed to be fluroxypyr resistant based on change in height (Figure 2a) and percent survival (Figure 2b) at 30 days after treatment (DAT), with 75% survival up to 628 g ae ha^−1^ of fluroxypyr (Figure 2b). Flur‐R was approximately 40 times more resistant than the susceptible population J01‐S and 36 times more resistant than 9,425 (Table 1). The population 9,425, which was previously reported to be fluroxypyr resistant (LeClere et al., 2018) was subsequently shown to have weak fluroxypyr resistance (Wu et al., 2020). Our results show 9,425 had less than 25% survival at 157 g ae ha^−1^ fluroxypyr (Figure 2b) and had a similar reduction in height as the known susceptible population J01‐S (Figure 2a). The LD_50_ ratios for J01‐S and 9,425 were not statistically different from 1, indicating that 9,425 is not resistant to fluroxypyr at field doses. Furthermore, Flur‐R was susceptible to dicamba (Figure 2c), with 8% survival at 70 g ae ha^−1^ and an LD_50_ of 56 g ae ha^−1^. Flur‐R is approximately seven times more susceptible than 9,425 to dicamba (Table 1).

### 3.2 Glyphosate, atrazine, and Chlorsulfuron single dose screening

No Flur‐R or J01‐S individuals survived glyphosate (870 g ae ha^−1^) or atrazine (2,240 g ai ha^−1^) treatments; however, 94% ( ±0.5%) of the Flur‐R population and 7% ( ±0.5%) of J01‐S individuals survived chlorsulfuron (137 g ai ha^−1^) indicating that there is multiple resistance in the Flur‐R population. Two target site mutations were identified in a SNP analysis of RNA‐sequencing data that confer ALS resistance, including a proline 197 to threonine mutation and a tryptophan 574 to leucine mutation in the *ALS* gene (Tranel & Wright, 2002) (Figure S1).

### 3.3 KASP

**FIGURE 3:**
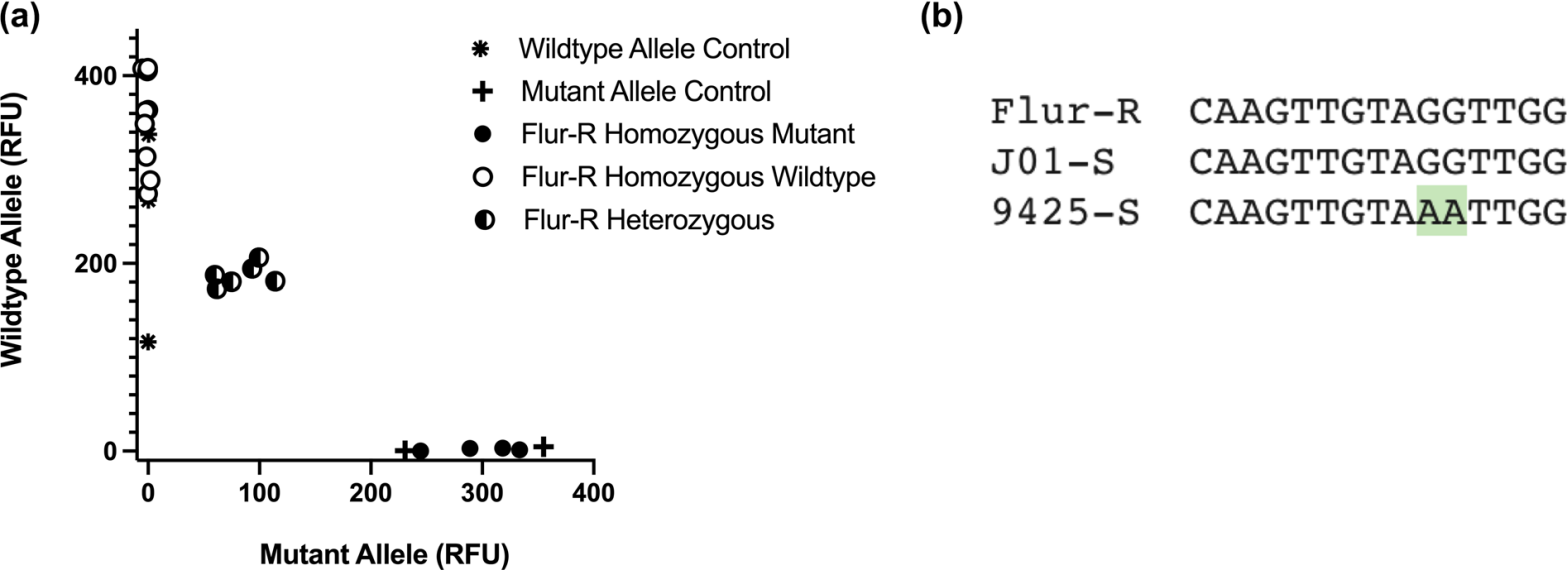
(a) KASP assay with fluroxypyr‐resistant individuals sprayed with 157 g ai ha^−1^ fluroxypyr. Wildtype allele control lines were fluroxypyr and dicamba susceptible line 7,710 and J01‐S. Mutant allele control was homozygous dicamba‐resistant line 9,425. (b) Consensus sequenced based on KASP and previously published sequencing data at the mutation point of interest in J01‐S, Flur‐R, and dicamba‐resistant line 9,425 conferring resistance to dicamba as reported by LeClere et al., 2018. The mutation from GG to AA confers a G127N change.

Kompetitive allele‐specific PCR (KASP) was used to genotype individuals using allelic discrimination to determine whether or not Flur‐R individuals contained the G127N *IAA16* mutation reported by LeClere et al. (2018). Specific fluorophore sequences were assigned to each forward primer, which generated a fluorescent signal to determine which allele was present in the kochia DNA sample. Relative Fluorescence Units (RFU) were measured to determine which of the fluorophore sequences amplified for each sample (Figure 3a). Of the 20 verified fluroxypyr‐resistant individuals tested from the Flur‐R population, 10 individuals displayed high RFU for the HEX labeled primer, indicating they had a homozygous wildtype genotype. There were six individuals that displayed approximately equal RFU for both alleles, indicating heterozygous individuals for G127N *IAA16*. Two known susceptible wild type controls were included (kochia lines J01‐S, 7710), as well as one homozygous mutant resistant control (9425). These results indicate that the asparagine‐127 *IAA16* mutant allele is not essential for fluroxypyr resistance, as most individuals were homozygous for the wildtype glycine‐127 *IAA16* allele and were resistant to fluroxypyr. The dicamba resistance asparagine‐127 *IAA16* allele is present and segregating in the Flur‐R population.

### 3.4 Fluroxypyr absorption, translocation, and metabolism

**FIGURE 4:**
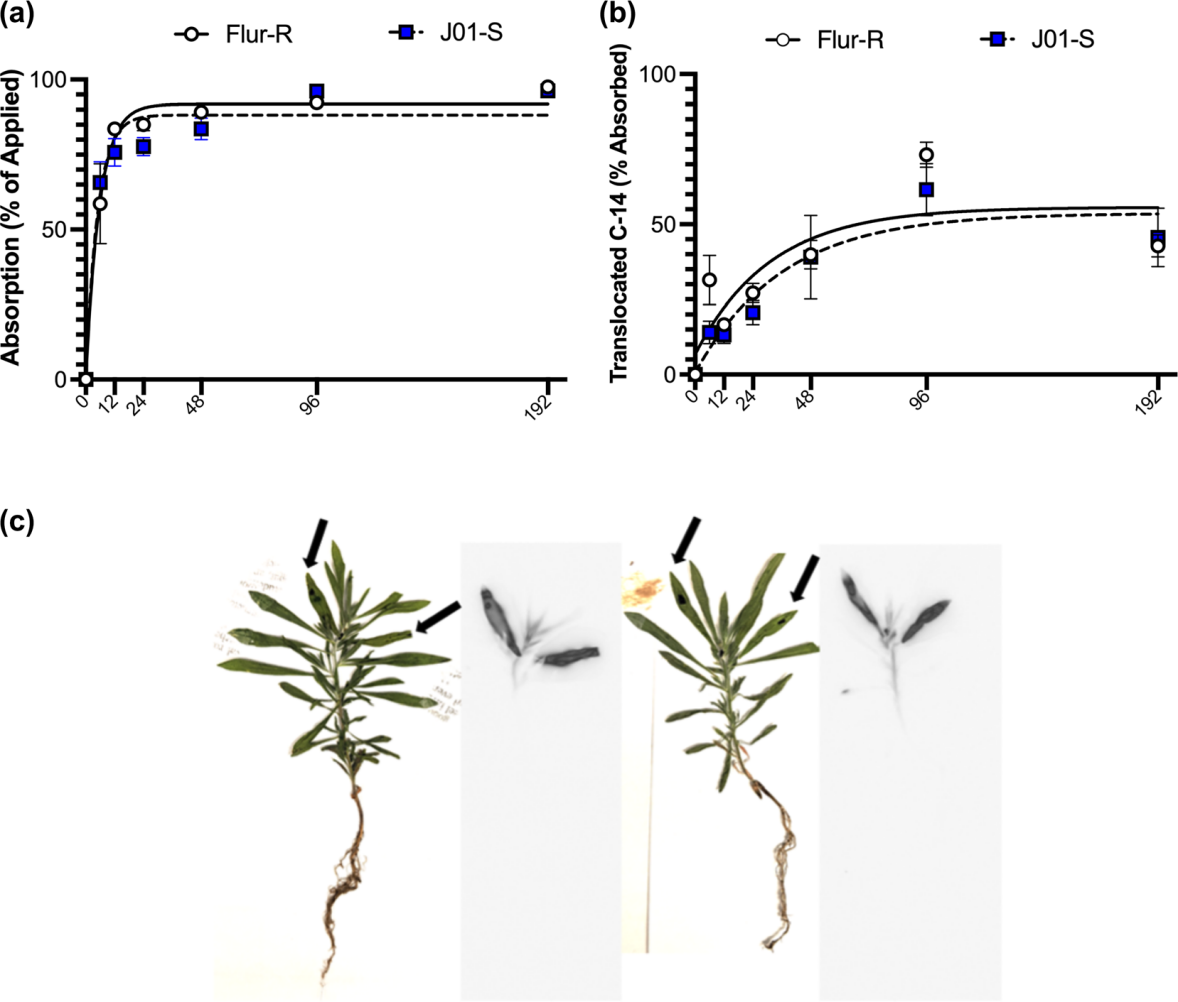
Whole plant absorption (a) and translocation (b) of fluroxypyr on resistant Flur‐R line and susceptible line J01‐S assessed over 6, 12, 24, 48, 96, and 192 h after treatment with [^14^C]‐fluroxypyr ester. The absorption and translocation graphs depict mean percent absorption as percent of applied radiation and mean percent translocation as percent of absorbed radiation to account for slight variation in application rates, with error bars representing SEM. There were no differences in absorption or translocation of [^14^C]‐fluroxypyr ester between Flur‐R and J01‐S. C. Pressed plant and phosphor‐images showed translocation of [^14^C]‐fluroxypyr ester in Flur‐R (left) and J01‐S (right) at 12 h, the time at which max absorption was at 90% in both lines. The black arrows mark the two treated meristem leaves on each individual. The phosphor image to the right of each pressed plant photo shows early‐stage translocation of [^14^C]‐fluroxypyr ester.

Mean percent radioactivity recovery was 82.99% (±3.39), with two samples per line at 192 h that were ≥50%. Maximum percent absorption of ~3.33 kBq [^14^C]‐fluroxypyr ester was 91.99% ( ±3.14) for Flur‐R, and 85% ( ±3.13) for J01‐S. No significant differences in maximum absorption between Flur‐R and J01‐S were found (pvalue = .155) (Figure 4a). The time (h) after treatment in which 90% of the herbicide is absorbed based on the model in R was not statistically different between 12 h ( ±2.15) for Flur‐R, and 9.7 h ( ±2.34) for J01‐S (pvalue = .47). There were no differences in translocation of [^14^C]‐fluroxypyr ester from the treated leaf to the rest of the plant between Flur‐R and J01‐S (Figure 4b,c).

**FIGURE 5:**
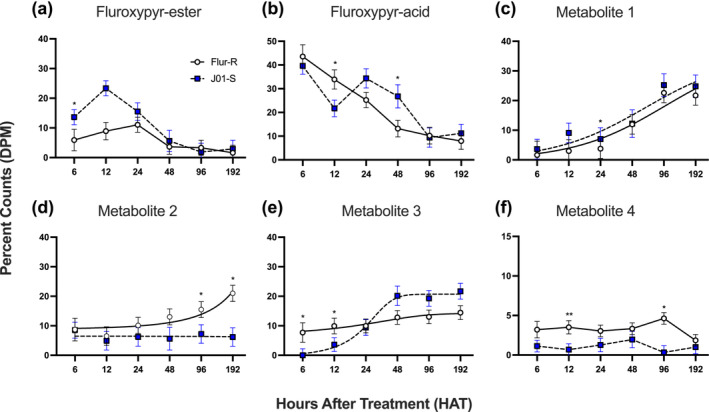
HPLC fluroxypyr parent and metabolite profiles over a 192 h time‐course in fluroxypyr resistant kochia (*Bassia scoparia)* line Flur‐R and fluroxypyr susceptible J01‐S. (a) Parent compound, [^14^C]‐fluroxypyr ester (9.5 min retention). (b) Biologically active compound, [^14^C]‐fluroxypyr acid (7.7 min retention). (c) Unknown metabolite 1 (4.5 min retention). (d) Unknown metabolite 2 (5.8 min retention). E. Unknown metabolite 3 (6.4 min retention). F. Unknown metabolite 4 (7.2 min retention). * *p* < .05, ** *p* ≤ .005, error bars represent SEM.

Whole plant metabolites were extracted for [^14^C]‐fluroxypyr ester metabolism studies. Analysis of the proportion of [^14^C]‐fluroxypyr ester in each population showed a significant difference at 12 h. The overall proportion of [^14^C]‐fluroxypyr ester was lower in Flur‐R than J01‐S, supporting rapid conversion from the [^14^C]‐fluroxypyr ester to biologically active [^14^C]‐fluroxypyr acid or other [^14^C]‐fluroxypyr metabolites (Figure 5). In Flur‐R, the high amount of [^14^C]‐fluroxypyr acid at 12 h was significantly reduced by 48 h, showing rapid conversion to other fluroxypyr metabolites (Figure 5b). At 96 h and 192 h, the proportion of unknown metabolites numbered 4 and 2 were higher or uniquely present in Flur‐R compared to J01‐S (Figure 5d,f). This suggests that the formation and flux of these metabolites are catalyzed by a process that is more active in Flur‐R than J01‐S and may play a role in reducing concentrations of phytotoxic [^14^C]‐fluroxypyr acid.

### 3.5 Differential expression analysis

**FIGURE 6:**
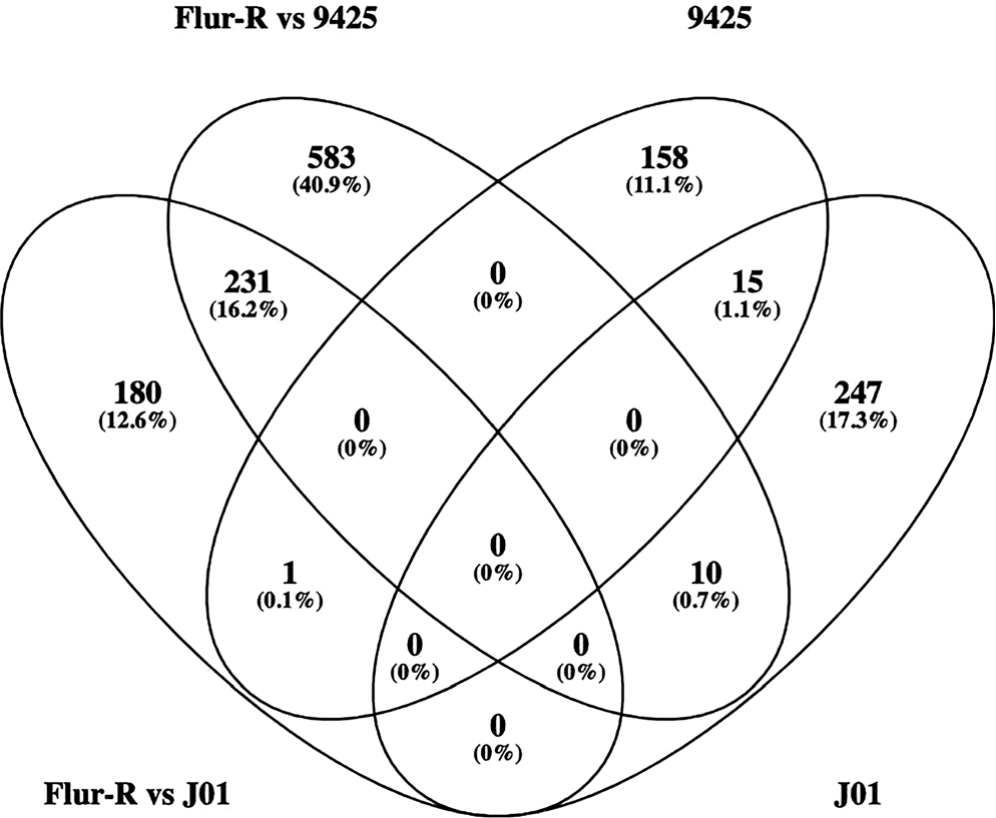
Venn diagram of upregulated genes between the untreated condition in Flur‐R compared to both untreated conditions in fluroxypyr susceptible lines 9,425‐S and J01‐S in DESeq2 (Flur‐R vs 9,425; Flur‐R vs J01). Genes upregulated in both 9,425‐S and J01‐S compared to Flur‐R in DESeq2 are represented by their singular line name in the diagram (J01, 9,425). Overlapping ovals represent genes that are commonly expressed at the untreated condition between comparisons.

We identified 231 unique genes that had higher expression in Flur‐R compared to both 9,425 and J01‐S at the untreated timepoint (Figure 6). Because we identified differential metabolism in Flur‐R, we explored the hypothesis that genes related to herbicide metabolism may have differential regulation or be highly expressed at the untreated timepoint in Flur‐R. Of these 231 highly expressed genes in Flur‐R at the untreated timepoint, there were six ABC transporters of both class B and G, including genes homologous to *ABCG31‐like* (*Bs.00 g217020.m01*), two similar ABCB28 annotated genes (*Bs.00 g454440.m01, Bs.00 g282300.m01*), two isoforms of *ABCG34* (*Bs.00 g184080.m01, Bs.00 g184080.m02*), and *ABCG29* (*Bs.00 g251290.m01*). There were five CYP450 annotated genes between two families, the CYP71 family (*CYP82D47* [*Bs.00 g486870.m01*], *CYP96A15* [*Bs.00 g541440.m01*], *CYP71D10/11* [*Bs.00 g051830.m01*], Ent‐kaurene oxidase [*Bs.00 g184110.m01*],) and the CYP85 family (*CYP90C1/D1* [*Bs.00 g245700.m01*]). Several types of glucosyltransferases were expressed, such as UDP‐glucosyltransferase 73B2 (*Bs.00 g142060.m01*), two isoforms of UDP‐glucuronosyl/UDP‐glucosyltransferase 89A2‐like (*Bs.00 g480980.m01, Bs.00 g480980.m02*), and UDP‐glycosyltransferase 87A1 (*Bs.00 g061050.m01*) (Table 2).

**TABLE 2:** Genes with higher expression at the untreated timepoint in kochia ( *Bassia scoparia* ) line Flur‐R compared to 9,425 and J01‐S lines at the untreated timepoint. Raw normalized counts and Log2 fold change for highly expressed ABC transporters, UDP glucosyltransferases, and cytochrome P450 monooxygenases in the fluroxypyr‐resistant population Flur‐R compared to either susceptible population 9,425 or J01‐S. Genes that are higher expressed in Flur‐R compared to both susceptible populations and are denoted with † and represented with the normalized count and fold change comparison to 9,425. Log2 fold change was calculated in DESeq2, log2 fold change standard error, and adjusted *p*‐value were also calculated in DESeq2. The Wald‐test obtained *p*‐values were adjusted using the Benjamini‐Hochberg method. The FDR was < .05.

Gene ID	Mean of normalized counts		Fold change	Log2 fold change ( ±SE)	*p*‐value (adjusted)	Gene description
	Flur‐R	9,425				
Bs.00 g184080.m02	2,258	3	753	9.70 (.75)	1.85E‐32	ABC‐G 34 isoform 2
Bs.00 g184080.m01	2,147	3	716	9.63 (.75)	9.86E‐32	ABC‐G 34 isoform 1
Bs.00 g217020.m01 †	63	0	63	8.98 (2.85)	1.31E‐06	ABC‐G 31‐like
Bs.00 g142060.m01	38	0	38	8.02 (2.81)	6.52E‐05	UDP‐glucosyltransferase 73B2 related
Bs.00 g184110.m01	133	2	67	6.24 (.96)	3.59E‐08	CYP701 subfamily (Ent‐ Kaurene oxidase)
Bs.00 g282300.m01 †	108	6	18	5.18 (1.47)	.0028	ABC‐G 28‐like
Bs.00 g480980.m01 †	510	14	36	5.01 (.63)	2.11E‐11	UDP‐glucuronosyl/UDP‐glucosyltransferase 89A2‐like
Bs.00 g480980.m03 †	538	15	36	4.99 (.63)	2.72E‐11	UDP‐glucuronosyl/UDP‐glucosyltransferase 89A2‐like
Bs.00 g541440.m01	232	10	23	4.21 (.72)	2.00E‐06	CYP96A15
Bs.00 g051830.m01	233	5	47	4.03 (1.48)	.0231	CYP71D10/11
Bs.00 g454440.m01 †	276	52	5	2.28 (.44)	.0003	Putative ABC‐B 28‐like
Bs.00 g061050.m01	6,018	1,187	5	2.08 (.59)	.027	UDP‐glycosyltransferase 87A1 related
Bs.00 g251290.m01	1,462	341	4	1.98 (.38)	.0008	ABC‐G 29‐like
Bs.00 g245700.m01	417	114	4	1.72 (.40)	.0113	CYP90C1/D1 (3‐Epi‐6‐Deoxocathasterone 23‐monooxygenase)

	Flur‐R	J01‐S				
Bs.00 g486870.m01	1847	61	31	4.85 (.55)	1.29E‐13	CYP82D47‐like
Bs.00 g142720.m01	8,246	3,606	2	1.17 (.15)	.0001	7‐deoxyloganetin glucosyltransferase‐like 85A23

**FIGURE 7:**
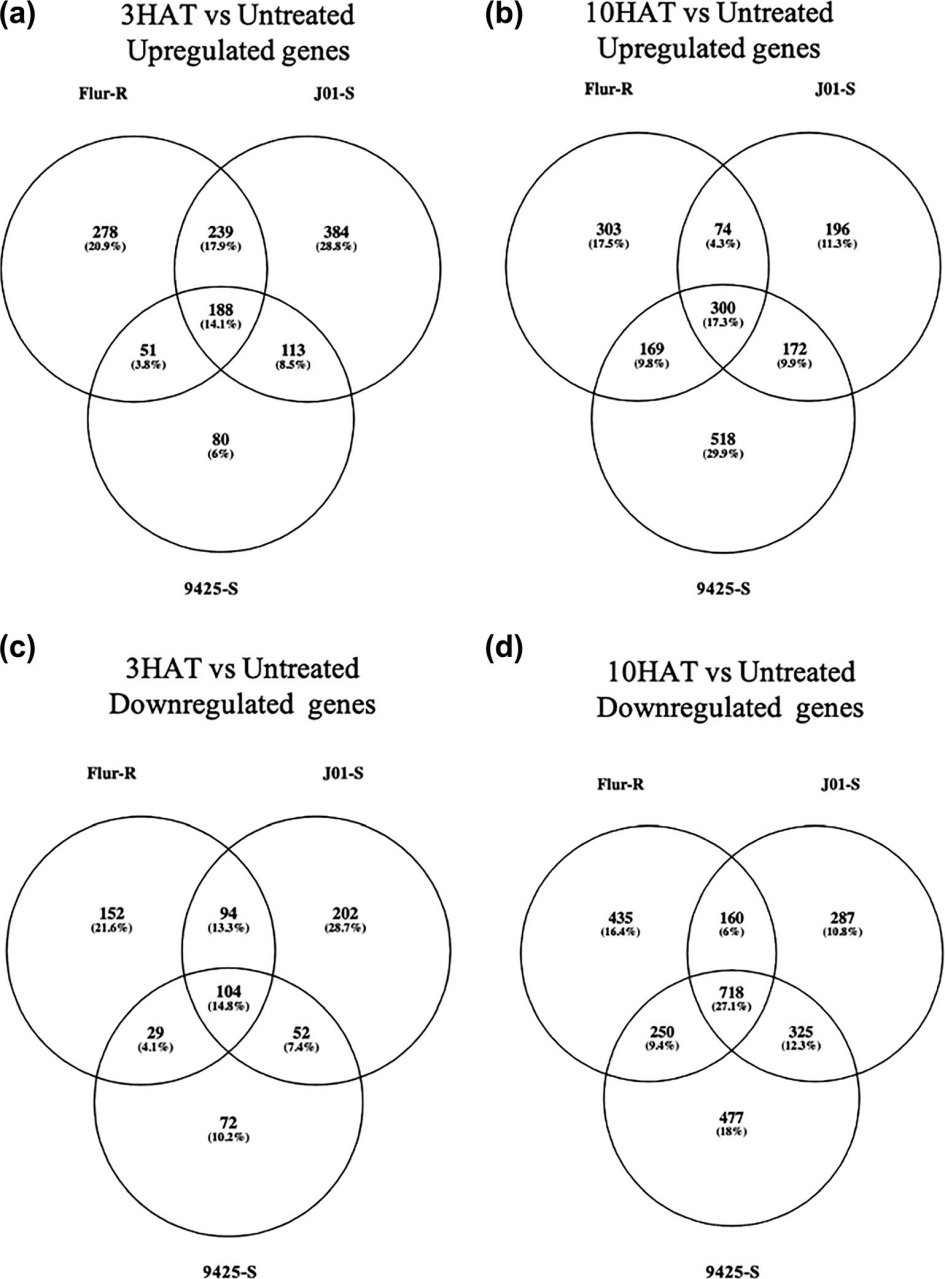
Number of transcripts that were either up or downregulated between the untreated condition and either 3 or 10 h after treatment (HAT) with fluroxypyr in fluroxypyr‐resistant line Flur‐R and susceptible lines 9,425 and J01‐S. (a) Shared and uniquely upregulated genes at 3 HAT among and between all three lines. (b) Shared and uniquely upregulated genes at 10 HAT among and between all three lines. (c) Shared and uniquely downregulated genes at 3 HAT among and between all three lines. (d) Shared and uniquely downregulated genes at 10 HAT among and between all three lines.

**FIGURE 8:**
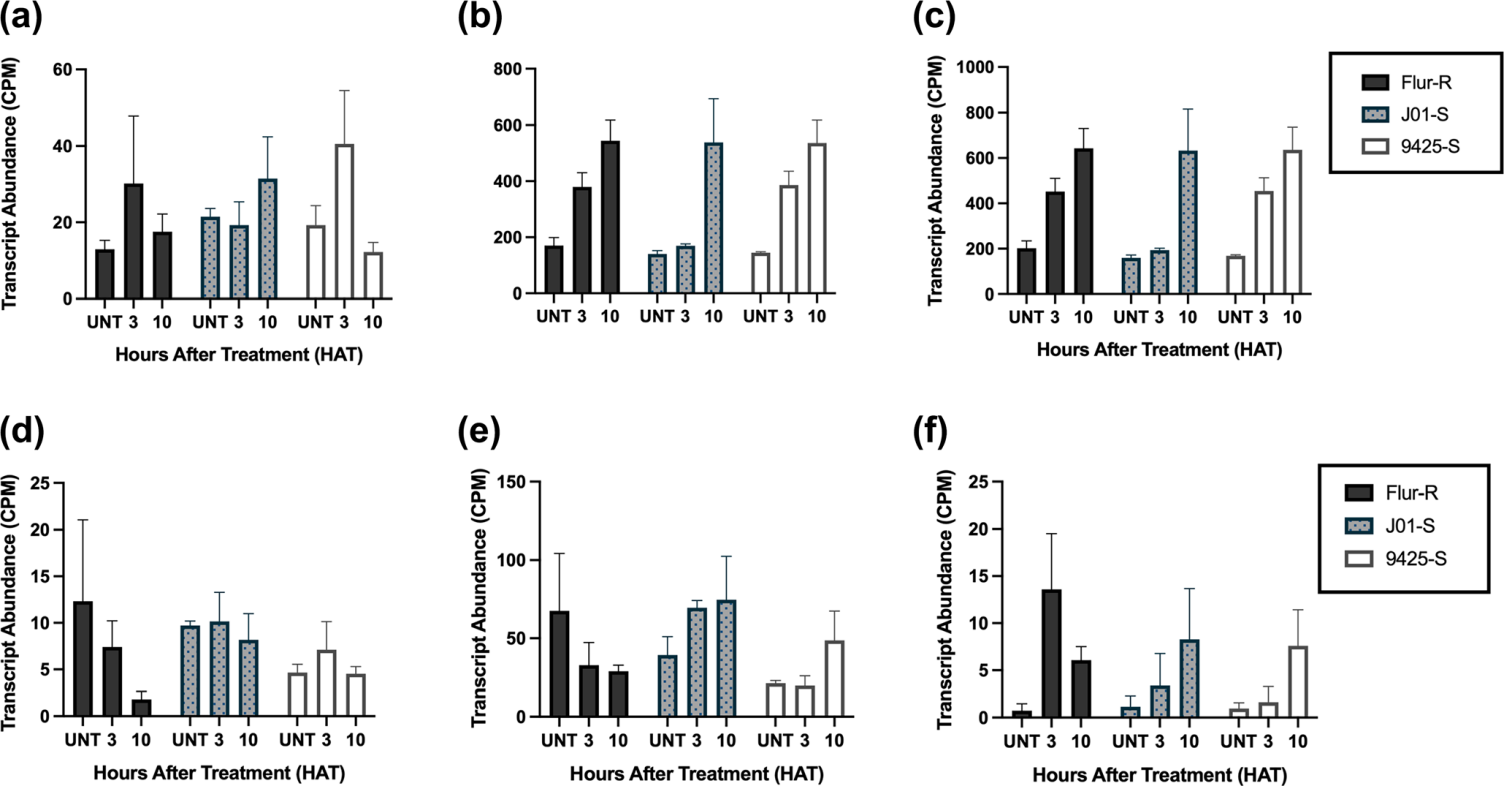
Expression profiles for auxin‐induced influx and efflux transporters and NCED in fluroxypyr‐resistant kochia ( *Bassia scoparia* ) Flur‐R, susceptible J01‐S, and susceptible 9,425 following differential expression analysis of RNA‐Seq data. (a) Auxin transporter‐like protein 2 (AUX1/LAX2/3) (Bs.00 g309300.m01). (b) Auxin efflux carrier component (Bs.00 g190770.m01). (c) Auxin efflux carrier component (Bs.00 g190770.m02). (d) NCED2 (Bs.00 g024060.m01) E. NCED2 (Bs.00 g059470.m01) F. NCED6 (Bs.00 g347670.m01). X‐axis treatments: untreated, 3 h after treatment (HAT), and 10 HAT grouped by kochia line. Normalized counts on the y‐axis were a result of the DESeq2 function and model fitting in R package “DESeq2”. Both isoforms of the auxin efflux carrier component were upregulated in response to fluroxypyr in the 9,425 line. There were no differences in expression for the AUX/LAX transporter. NCED6 was induced at both 3 HAT and 10 HAT in Flur‐R, while NCED2 was downregulated in Flur‐R.

When analyzing the treated timepoints within Flur‐R, J01‐S, and 9,425, 188 (3 h after treatment [HAT] vs untreated) and 300 genes (10 HAT vs untreated) were upregulated in response to fluroxypyr treatment in all three lines (Figure 7a,b). Of those shared upregulated genes, auxin‐responsive genes encoding proteins such as GH3.2 (*Bs.00 g477580.m01*), Ethylene responsive transcription factors, Small auxin‐up RNAs (SAURs), Aux/IAAs, and ACS (*Bs.00 g478760.m01*) were among them, indicating that all three kochia lines perceived fluroxypyr and had transcriptional activation of these auxin‐responsive genes following fluroxypyr treatment (Figure S2). The Ethylene responsive transcription factors, *GH3*, and *ACS* were in the top 20 genes with the highest fold change through the 3 HAT vs untreated and 10 HAT vs untreated timepoints in Flur‐R, J01‐S, and 9,425 (Tables 3, 4, and 5). Two isoforms of the IAA cellular transporter PIN were upregulated in 9425 at 10 HAT (*Bs.00 g190770.m01* and *Bs.00 g190770.m02*), but the response in Flur‐R and J01‐S did not meet the differential expression filtering criteria and therefore the response was not statistically different following fluroxypyr treatment (Figure 8). Within the Flur‐R line at 3 HAT vs untreated, there were 278 uniquely upregulated genes and 303 at 10 HAT vs untreated (Figure 7a,b). Some unique auxin‐induced genes such as *SAURs* and *ARF11* were upregulated in Flur‐R, but six additional ABC transporters of class G, two ABC transporters of class C, one ABC transporter of class A, six additional UDP‐glucosyltransferases (GTs), and three sugar transporters were upregulated following fluroxypyr treatment (expression data not shown). CYP450s *CYP81B2* (*Bs.00 g431990.m01*), *CYP82D47*, and *CYP71A9* (*Bs.00 g241110.m01*) were induced by fluroxypyr treatment, as well as four Glutathione s‐transferases (GSTs) in Flur‐R at 3 and 10 HAT compared to the untreated timepoint.

**TABLE 3:** Top 20 upregulated genes in fluroxypyr‐resistant kochia ( *Bassia scoparia* ) line Flur‐R at 3 h after treatment (HAT) and 10 HAT compared to the untreated timepoint. fold change was calculated using the mean of normalized counts, which was produced using the DESeq2 package in R. Log2 fold change was calculated in DESeq2, log2 fold change standard error and adjusted *p*‐value were also calculated in DESeq2. The Wald‐test obtained *p*‐values were adjusted using the Benjamini‐Hochberg method. The FDR was < .05.

Gene ID	Mean of normalized counts		Fold change	Log2 fold change ( ±SE)	*p*‐value	Gene description
	Flur‐R untreated	Flur‐R 3HAT				
Bs.00 g016210.m01^j^, ^†^	.71	607	855	5.30 (.39)	1.59E‐14	Precursor of CEP13/CEP14
Bs.00 g306100.m01	8	383	48	4.79 (.33)	2.84E‐28	Transcription factor, MADS‐box
Bs.00 g477580.m01^c^, ^†^				4.63 (.40)	1.22E‐35	GH3 family protein
Bs.00 g523550.m01	20	1,131	57	4.57 (.380	8.34E‐24	Reverse transcriptase zinc‐binding domain
Bs.00 g418990.m01	34	1,534	45	4.48 (.37)	3.11E‐23	Ethylene‐responsive transcription factor
Bs.00 g010340.m01	1,187	24,154	20	4.43 (.28)	3.94E‐42	Membrane attack complex component/perforin (MACPF) domain
Bs.00 g435130.m01	34	1,425	42	4.11 (.39)	2.77E‐19	Proton‐dependent oligopeptide transporter family
Bs.00 g315820.m01	242	7,163	30	4.03 (.37)	2.01E‐18	Amino acid transporter
Bs.00 g520970.m01	4	791	198	3.98 (.47)	3.34E‐13	Uncharacterized protein
Bs.00 g419000.m01^a^	4	1779	445	3.99 (.49)	3.20E‐11	Dehydration‐responsive element‐binding protein 1A‐related
Bs.00 g315840.m01	184	5,117	28	3.92 (.38)	1.28E‐16	Amino acid transporter
Bs.00 g301780.m01	650	24,203	37	3.90 (.40)	2.28E‐17	ABC transporter G family member 40
Bs.00 g087440.m01	17	547	32	3.88 (.40)	5.86E‐14	Amino acid transporter
Bs.00 g181270.m02^a^	150	5,353	36	3.87 (.42)	4.17E‐14	Protein NLP6‐related
Bs.00 g257560.m01	1	330	330	3.85 (.45)	3.93E‐11	C2 domain (calcium/lipid‐binding domain, Calb)
Bs.00 g200680.m01	1	80	80	3.85 (.42)	2.58E‐07	Uncharacterized protein
Bs.00 g244620.m01	75	1873	25	3.85 (.34)	7.44E‐20	Uncharacterized protein
Bs.00 g301770.m01	65	2,253	35	3.82 (.40)	3.74E‐16	ABC transporter G family member 40
Bs.00 g428240.m01	3	126	42	3.78 (.42)	3.22E‐10	Extended Synaptotagmin‐related
Bs.00 g036810.m01	750	16,564	22	3.73 (.36)	1.72E‐17	Protein phosphatase 2C

	Flur‐R untreated	Flur‐R 10 HAT				
Bs.00 g016210.m01^j^, ^†^	.71	2,485	4,007	7.43 (.36)	9.28E‐23	Precursor of CEP13/CEP14
Bs.00 g477580.m01^c^, ^†^	67	29,708	443	6.01 (.41)	6.01E‐57	GH3
Bs.00 g239120.m01^k^	7	1,135	162	5.82 (.39)	1.82E‐33	Aquaporin transporter
Bs.00 g168520.m01^k^	35	3,260	93	5.25 (.35)	1.44E‐36	Cold regulated protein 27
Bs.00 g168520.m02^d^	37	3,298	89	5.41 (.38)	1.41E‐43	Cold regulated protein 27
Bs.00 g107600.m01	13	2,990	230	5.21 (.43)	7.35E‐34	Barwin‐like endoglucanases
Bs.00 g370370.m01	3	495	65	5.14 (.41)	1.50E‐22	Ethylene‐responsive transcription factor 13 related
Bs.00 g431740.m01	33	4,437	134	5.12 (.44)	2.94E‐27	Heme‐dependent peroxidases
Bs.00 g057300.m01	.34	142	418	5.06 (.49)	3.98E‐08	CYP71D10‐like
Bs.00 g217150.m01	6	1,485	248	4.92 (.44)	4.39E‐28	Bet v I/major latex protein
Bs.00 g122020.m01	42	16,883	393	4.88 (033)	2.86E‐36	Uncharacterized protein
Bs.00 g261130.m01	43	6,160	143	4.83 (.33)	1.71E‐23	Bet v I/major latex protein
Bs.00 g291860.m01	0	153	153	4.78 (.47)	7.06E‐11	Secoisolariciresinol dehydrogenase
Bs.00 g478760.m01^h^	1	930	930	4.48 (.46)	5.42E‐19	1‐Aminocyclopropane‐1‐carboxylate synthase 4 related
Bs.00 g282410.m01	6	730	122	4.43 (.45)	2.22E‐19	Cysteine‐rich repeat secretory protein 38
Bs.00 g056520.m01	532	16,588	31	4.39 (.33)	5.82E‐31	Alanine dehydrogenase/pyridine nucleotide transhydrogenase
Bs.00 g370420.m01	.37	76	205	4.35 (.45)	5.86E‐07	Uncharacterized protein
Bs.00 g422990.m01^d^	4,681	137,203	29	4.28 (.32)	4.34E‐32	4‐Hydroxyphenylpyruvate dioxygenase‐like
Bs.00 g020740.m01	5	709	142	4.27 (.48)	1.40E‐16	WRKY transcription factor
Bs.00 g148640.m01^m^	1,158	32,325	28	4.26 (.30)	7.67E‐36	2‐Oxoisovalerate dehydrogenase subunit alpha 2

^a^ Shared between J01‐S 3 HAT and Flur‐R 3 HAT top 20 upregulated genes.

^c^ Shared between 9,425‐S 10 HAT, J01‐S 10 HAT and Flur‐R 3/10 HAT top 20 upregulated genes.

^d^ Shared between J01‐S 10 HAT and Flur‐R 3 HAT upregulated top 20 upregulated genes.

^h^ Shared between 9,425‐S 3 HAT, Flur‐R 10 HAT, and J01‐S 10 HAT top 20 upregulated genes.

^j^ Shared between 9,425‐S 10 HAT, Flur‐R 3 HAT/10 HAT, and J02‐S 10 HAT top 20 upregulated genes.

^k^ Shared between 9,425‐S 10 HAT, Flur‐R 10 HAT, and J01‐S 10 HAT top 20 upregulated genes.

^m^ Shared between 9,425‐S 10 HAT and Flur‐R 10 HAT top 20 upregulated genes.

^†^ Shared between Flur‐R 3 HAT/10 HAT top 20 upregulated genes.

**TABLE 4:** Top 20 upregulated genes in fluroxypyr‐susceptible kochia ( *Bassia scoparia* ) line J01‐S at 3 h after treatment (HAT) and 10 HAT compared to the untreated timepoint. fold change was calculated using the mean of normalized counts which was produced using the DESeq2 package in R. Log2 fold change was calculated in DESeq2, log2 fold change standard error and adjusted *p*‐value were also calculated in DESeq2. The Wald‐test obtained *p*‐values were adjusted using the Benjamini‐Hochberg method. The FDR was <.05.

Gene ID	Mean of normalized counts		Fold change	Log2 fold change ( ±SE)	*p*‐value	Gene description
	J01‐S untreated	J01‐S 3 HAT				
Bs.00 g058350.m01	4	3,428	763	7.33 (.40)	2.01E‐40	NADH oxidoreductase‐related
Bs.00 g144030.m01	173	40,872	237	6.42 (.44)	1.39E‐37	Glycoside hydrolase, family 16
Bs.00 g487370.m01	3	694	247	5.84 (.46)	1.66E‐17	Alpha/Beta hydrolase fold
Bs.00 g397110.m01	0	494	494	5.46 (.51)	1.71E‐09	Zinc finger, RING/FYVE/PHD‐type
Bs.00 g435120.m01	31	2,720	87	5.44 (.37)	2.45E‐36	Proton‐dependent oligopeptide transporter family
Bs.00 g419000.m01^a^	0	762	762	5.43 (.53)	4.51E‐10	AP2/ERF
Bs.00 g122020.m01^†^	20	10,546	515	5.43 (.46)	4.17E‐37	Uncharacterized protein
Bs.00 g142660.m01	232	13,906	60	5.39 (.32)	6.56E‐47	Exordium‐like
Bs.00 g430680.m01	5	8,380	1847	5.38 (.53)	2.75E‐25	Protein phosphatase 2C family
Bs.00 g167370.m01	23	2,527	110	5.23 (.48)	2.31E‐18	Elo, fatty acid acyl transferase‐related
Bs.00 g058830.m01	337	54,400	162	5.20 (.50)	5.41E‐20	Harbinger transposase‐derived nuclease domain
Bs.00 g415260.m01	328	31,294	95	5.12 (.46)	4.78E‐21	WRKY domain
Bs.00 g361960.m01	24	1,628	67	4.96 (.43)	1.07E‐20	Gibberellin 2‐Beta‐dioxygenase 4
Bs.00 g428250.m02	34	5,275	154	4.87 (.52)	7.13E‐16	C2 domain (calcium/lipid‐binding domain, Calb)
Bs.00 g244130.m01	100	15,352	154	4.70 (.51)	3.15E‐17	Protein TIFY 11A‐related
Bs.00 g181270.m02^a^	74	6,520	88	4.65 (.49)	2.26E‐16	Uncharacterized protein
Bs.00 g428250.m01	34	4,864	142	4.64 (.53)	5.42E‐14	Extended Synaptotagmin‐related
Bs.00 g512260.m01^i^	552	20,157	37	4.63 (.42)	9.41E‐18	Glyoxalase/Fosfomycin resistance/dioxygenase domain
Bs.00 g228950.m01	2	333	207	4.56 (.55)	1.44E‐07	Dehydration‐responsive element‐binding protein 1a‐related
Bs.00 g481180.m01	11	762	70	4.55 (.46)	1.44E‐15	Malectin‐like carbohydrate‐binding domain

	J01‐S untreated	J01‐S 10 HAT				
Bs.00 g122020.m01^†^	20	13,981	683	6.76 (.41)	3.41E‐61	Uncharacterized protein
Bs.00 g016210.m01^j^	2	1874	811	6.29 (.43)	2.46E‐24	Precursor of CEP13/CEP14
Bs.00 g176460.m01	7	1,155	173	5.88 (.39)	3.91E‐32	At‐hook motif nuclear‐localized protein 27
Bs.00 g218880.m01	3	529	183	5.30 (.43)	3.47E‐17	D‐Arabinono‐1,4‐lactone oxidase
Bs.00 g168520.m01^k^	43	3,475	80	5.23 (.40)	6.91E‐31	Cold regulated protein 27
Bs.00 g168520.m02^k^	42	3,334	78.7	5.08 (.42)	1.54E‐27	Cold regulated protein 27
Bs.00 g176480.m01	1	295	511	5.07 (.46)	6.62E‐07	At‐hook motif nuclear‐localized protein 27
Bs.00 g239120.m01^k^	6	1,088	176	4.96 (.49)	3.49E‐18	Aquaporin Tip3–1‐related
Bs.00 g478760.m01^h^	2	793	457	4.70 (.49)	3.96E‐14	1‐Aminocyclopropane‐1‐carboxylate synthase 4‐related
Bs.00 g112620.m01	7	731	110	4.56 (.47)	8.44E‐17	Lipase/Lipooxygenase domain
Bs.00 g044610.m01^l^	263	9,925	37.7	4.54 (.35)	1.05E‐29	Uncharacterized protein
Bs.00 g304090.m01^g^	154	4,039	26.3	4.28 (.31)	7.03E‐32	AP2/ERF domain
Bs.00 g477580.m01^c^	279	45,370	162	4.28 (.50)	7.01E‐23	Indole‐3‐acetic acid‐Amido Synthetase GH3.2‐related
Bs.00 g112710.m01	416	34,778	83.5	4.24 (.48)	7.24E‐20	Lipoxygenase, C‐terminal
Bs.00 g275080.m01	2	376	228	4.23 (.48)	5.48E‐11	Heme‐dependent peroxidases
Bs.00 g364070.m01	380	9,639	25.4	4.22 (.36)	2.28E‐22	NAC domain‐containing protein 10‐related
Bs.00 g422990.m01^d^	3,600	120,541	33.5	4.15 (.40)	8.07E‐20	4‐Hydroxyphenylpyruvate dioxygenase
Bs.00 g359220.m02^n^	12	14,262	1,145	4.10 (.58)	4.71E‐22	Proteinase inhibitor I13
Bs.00 g520060.m01	448	8,022	17.9	3.93 (.24)	1.18E‐46	B‐box domain protein 26‐related
Bs.00 g058370.m01	1	152	131	3.92 (.47)	3.23E‐07	Metacaspase‐4‐related

^a^ Shared between J01‐S 3 HAT and Flur‐R 3 HAT top 20 upregulated genes.

^c^ Shared between 9,425‐S 10 HAT, J01‐S 10 HAT, and Flur‐R 3/10 HAT top 20 upregulated genes.

^d^ Shared between J01‐S 10 HAT and Flur‐R 10 HAT top 20 upregulated genes.

^g^ Shared between 9,425‐S 3 HAT and J01‐S 10 HAT top 20 upregulated genes.

^h^ Shared between 9,425‐S 3 HAT, Flur‐R10 HAT, and J01‐S 10 HAT top 20 upregulated genes.

^i^ Shared between J01‐S 3 HAT and 9,425‐S 3 HAT upregulated top 20.

^j^ Shared between 9,425‐S 10 HAT, Flur‐R 3/10 HAT, and J01‐S 3 HAT/10 HAT top 20 upregulated genes.

^k^ Shared between 9,425‐S 10 HAT, Flur‐R 10 HAT, and J01‐S 10 HAT top 20 upregulated genes.

^l^ Shared between 9,245‐S 3 HAT/10 HAT and J01‐S 10 HAT top 20 upregulated genes.

^n^ Shared between 9,425‐S 10 HAT and J01‐S 10 HAT top 20 upregulated genes.

^†^ Shared between J01‐S 3 HAT/10 HAT top 20 upregulated genes.

**TABLE 5:** Top 20 upregulated genes in fluroxypyr‐susceptible kochia ( *Bassia scoparia* ) line 9,425 at 3 h after treatment (HAT) and 10 HAT compared to the untreated timepoint. fold change was calculated using the mean of normalized counts which was produced using the DESeq2 package in R. Log2 fold change was calculated in DESeq2, log2 fold change standard error and adjusted *p*‐value were also calculated in DESeq2. The Wald‐test obtained *p*‐values were adjusted using the Benjamini‐Hochberg method. The FDR was <.05.

Gene ID	Mean of normalized counts		Fold change	Log2 fold change ( ±SE)	*p*‐value	Gene description
	9,425 untreated	9,425 3 HAT				
Bs.00 g044610.m01^l^ ^†^	188	9,324	50	4.69 (.32)	3.31E‐39	Uncharacterized protein
Bs.00 g301680.m01	973	17,616	18	3.73 (.27)	8.83E‐32	Auxin‐responsive protein IAA15
Bs.00 g174320.m01^†^	39	664	17	3.36 (.31)	6.95E‐19	Histidine kinase/HSP90‐like ATPase superfamily
Bs.00 g050510.m01	150	1971	13	3.36 (.26)	1.40E‐24	Cytochrome P450 734A1
Bs.00 g229060.m01	84	2,342	28	3.34 (.41)	1.90E‐11	Multi antimicrobial extrusion protein/protein detoxification 50
Bs.00 g107340.m01	474	5,929	13	3.34 .24)	5.55E‐30	Auxin‐responsive protein IAA1‐related
Bs.00 g293750.m01	16	283	18	3.32 (.34)	6.03E‐14	Uncharacterized protein
Bs.00 g478760.m01^h^	7	583	83	3.31 (.44)	3.80E‐13	1‐Aminocyclopropane‐1‐carboxylate synthase 4‐related
Bs.00 g506330.m01	49	1,128	23	3.27 (.41)	4.61E‐10	Chaperone J‐domain superfamily
Bs.00 g044350.m01	605	9,804	16	3.14 (.36)	7.06E‐12	Uncharacterized protein
Bs.00 g512260.m01^i^	100	4,635	46	3.13 (.47)	1.83E‐05	Lactoylglutathione Lyase glyoxalase I
Bs.00 g305330.m01	79	2,260	29	3.06 (.45)	5.39E‐07	Cytochrome P450 76c1‐related
Bs.00 g357520.m01	2	88	41	2.96 (.42)	2.21E‐07	Late embryogenesis abundant protein
Bs.00 g364640.m01	641	7,072	11	2.94 (.31)	4.70E‐13	Signal transduction response regulator, receiver domain
Bs.00 g304090.m01^g^	196	2097	11	2.91 (.30)	1.87E‐14	AP2/ERF transcription factor ERF/PTI6
Bs.00 g204690.m01	249	4,008	16	2.90 (.41)	1.21E‐07	Zinc finger, RING/FYVE/PHD‐type
Bs.00 g048560.m01	768	8,441	11	2.88 (.31)	4.70E‐13	Auxin‐responsive protein IAA29
Bs.00 g112660.m01	352	9,548	27	2.87 (.42)	7.59E‐10	Linoleate 9S‐lipoxygenase 5, Chloroplastic
Bs.00 g415260.m01	263	9,451	36	2.86 (.47)	3.22E‐06	WRKY transcription factor 46‐related
Bs.00 g290110.m01	336	3,706	11	2.85 (.35)	7.53E‐10	Uncharacterized protein

	9,425 untreated	9,425 10 HAT				
Bs.00 g427230.m01	28	5,143	183	6.20	1.09E‐37	Member of ‘GDXG’ family of Lipolytic enzymes
Bs.00 g168520.m01^k^	46	4,282	94	5.64	6.80E‐40	Cold regulated protein 27
Bs.00 g168520.m02^k^	53	4,276	81	5.44	1.36E‐35	Cold regulated protein 27
Bs.00 g174320.m01^†^	39	2,571	66	5.40	1.17E‐53	Histidine kinase/HSP90‐like ATPase superfamily
Bs.00 g044610.m01^l^, ^†^	188	10,766	57	5.07	3.35E‐49	Uncharacterized protein
Bs.00 g261820.m01	0	77	77	4.93	4.95E‐07	Pectin Lyase fold/virulence factor
Bs.00 g016210.m01^j^	2	1,675	988	4.87	9.19E‐22	Precursor of CEP13/CEP14
Bs.00 g413330.m01	620	71,783	116	4.85	4.01E‐22	Cystathionine gamma‐Lyase
Bs.00 g148640.m01^m^	660	27,633	42	4.71	1.26E‐34	2‐Oxoisovalerate dehydrogenase subunit alpha 2, mitochondrial
Bs.00 g305280.m01	0	52	52	4.65	1.41E‐05	Allergen V5/TPX‐1‐related, conserved site
Bs.00 g142300.m01	0	48	48	4.58	2.08E‐05	Pectin Lyase fold/virulence factor
Bs.00 g477660.m01	9	4,426	510	4.56	1.47E‐25	Gibberellin‐regulated GASA/GAST/SNAKIN family protein‐related
Bs.00 g359220.m02^n^	41	15,538	383	4.56	1.16E‐25	Proteinase inhibitor I13, potato inhibitor I superfamily
Bs.00 g239120.m01^k^	2	1,207	619	4.54	5.71E‐14	Aquaporin TIP3–1‐related
Bs.00 g208750.m01	287	11,553	40	4.52	1.72E‐25	CASP‐like protein 1E1‐related
Bs.00 g430360.m01	60	2,663	44	4.44	7.76E‐24	Protein early flowering 4
Bs.00 g100040.m01	3	789	228	4.43	5.81E‐09	Bet V I/major latex protein
Bs.00 g477580.m01^c^	92	39,708	431	4.41	8.98E‐30	Indole‐3‐acetic acid‐Amido Synthetase GH3.2‐related
Bs.00 g526000.m01	228	9,711	43	4.40	4.47E‐21	NAC domain
Bs.00 g447890.m01	0	73	73	4.36	6.43E‐07	Pectinesterase

^c^ Shared between 9,425‐S 10 HAT, J01‐S 10 HAT, and Flur‐R 3 HAT/10 HAT top 20 upregulated genes.

^g^ Shared between 9,425‐S 3 HAT and J01‐S 10 HAT top 20 upregulated genes.

^h^ Shared between 9,425‐S 3 HAT, Flur‐R 10 HAT, and J01‐S 10 HAT top 20 upregulated genes.

^I^ Shared between 9,425‐S 3 HAT and J01‐S 3 HAT top 20 upregulated genes.

^j^ Shared between 9,425‐S 10 HAT, Flur‐R 3 HAT/10 HAT, and J01‐S 3 HAT/10 HAT top 20 upregulated genes.

^k^ Shared between 9,425‐S 10 HAT, Flur‐R 10 HAT, and J01‐S 10 HAT top 20 upregulated genes.

^l^ Shared between 9,245‐S 3 HAT/10 HAT and J01‐S 10 HAT top 20 upregulated genes.

^m^ Shared between 9,425‐S 10 HAT and Flur‐R 10 HAT top 20 upregulated genes.

^n^ Shared between 9,425‐S 10 HAT and J01‐S 10 HAT top 20 upregulated genes.

^†^ Shared between 9,425‐S 3 HAT/10 HAT top 20 upregulated genes.

When the downregulated 3 and 10 HAT timepoints were contrasted with the untreated timepoint within each line, Flur‐R, J01‐S, and 9,425 identified 104 and 718 common fluroxypyr downregulated genes for the 3 and 10 HAT vs untreated timepoints, respectively (Figure 7c,d). Twelve of these shared genes were related to photosystem I and II at 10 HAT. Transcripts encoding key proteins related to photosynthetic electron transport such as Chlorophyll A‐B binding protein (*Bs.00 g240870.m01, Bs.00 g240870.m02*) and ATP synthase (*Bs.00 g432500.m01*) were downregulated in all three lines and were present in the top 20 downregulated genes for all three lines (Tables 6, 7, and 8). Two chlorophyll biosynthesis regulator genes encoding Early light‐induced protein‐1 (*Bs.00 g421070.m01, Bs.00 g420960.m01*) were uniquely downregulated and among the genes with the highest downregulation in both susceptible lines. These proteins play a role in preventing oxidative stress and excess accumulation of free chlorophyll (Hutin et al., 2003). Additionally, four Cellulose synthase genes (*Bs.00 g015170.m01, Bs.00 g015170.m02, Bs.00 g056700.m01, Bs.00 g260720.m01*) were downregulated in both susceptible lines and are of interest due to the role cellulose plays in cell wall structural support. Genes uniquely downregulated at 10 HAT in Flur‐R included two additional photosystem II subunit genes (*Bs.00 g059220.m01, Bs.00 g338570.m01*) as well as genes encoding several synthases such as Terpene synthase (*Bs.00 g074880.m01*), Polyprenyl synthetase (*Bs.00 g449610.m01*), Strictosidine synthase (*Bs.00 g057800.m01*), Phosphomethylpyrimidine synthase (*Bs.00 g253210.m01*), Aminodeoxychorismate (ADC) synthase (*Bs.00 g135570.m01*), and ABA biosynthesis gene *NCED2* (*Bs.00 g024060.m01*).

**TABLE 6:** Top 20 downregulated genes in fluroxypyr resistant kochia ( *Bassia scoparia* ) line Flur‐R at 3 h after treatment (HAT) and 10 HAT compared to the untreated timepoint. fold change was calculated using the mean of normalized counts which was produced using the DESeq2 package in R. Log2 fold change was calculated in DESeq2, log2 fold change standard error and adjusted *p*‐value were also calculated in DESeq2. The Wald‐test obtained *p*‐values were adjusted using the Benjamini‐Hochberg method. The FDR was <.05.

Gene ID	Mean of normalized counts		Fold change	Log2 fold change ( ±SE)	*p*‐value	Gene description
	Flur‐R untreated	Flur‐R 3 HAT				
Bs.00 g258890.m01^†^	136	2	−66	−3.96 (.44)	5.28E‐11	LRR
Bs.00 g104620.m01^e^, ^†^	7,356	172	−43	−3.64 (.43)	4.34E‐13	Protein kinase
Bs.00 g354480.m01^r^	4,017	217	−19	−3.35 (.37)	4.39E‐13	SAM dependent carboxyl methyltransferase
Bs.00 g370120.m01^r^	197	7	−29	−3.19 (.45)	2.63E‐08	Lipid binding domain
Bs.00 g362240.m01	574	29	−20	−3.16 (.42)	7.22E‐09	Bicarbonate transporter
Bs.00 g195790.m01^b^	234	10	−24	−3.10 (.44)	2.63E‐08	Proton dependent oligopeptide transporter
Bs.00 g056860.m01	127	1	−95	−3.01 (.48)	1.48E‐06	Peptidase/proteinase inhibitor I9
Bs.00 g119650.m01	1,565	66	−24	−2.97 (.44)	4.39E‐08	Nicotianamine synthase
Bs.00 g251440.m01^r^	687	51	−13	−2.97 (.37)	3.33E‐10	Multicopper oxidase
Bs.00 g126000.m01^†^	66,186	1,694	−39	−2.94 (.47)	3.76E‐08	NADH cytochrome B5 reductase
Bs.00 g123470.m01	475	38	−13	−2.92 (.37)	3.95E‐10	Glycoside hydrolase
Bs.00 g264170.m01^r^	5,895	687	−9	−2.87 (.23)	6.32E‐23	Glycoside hydrolase
Bs.00 g195800.m01	205	14	−15	−2.82 (.42)	3.44E‐07	Proton dependent oligopeptide transporter
Bs.00 g403960.m01^†^	36,087	2,902	−12	−2.75 (.39)	1.81E‐08	Carotenoid oxygenase
Bs.00 g528960.m01^r^	1,273	96	−13	−2.73 (.40)	1.24E‐07	Auxin‐inducible
Bs.00 g348080.m01	965	77	−13	−2.73 (.40)	9.58E‐08	Uncharacterized protein
Bs.00 g420960.m01^p^	1713	12	−148	−2.65 (049)	1.28E‐08	Chlorophyll A‐B binding protein
Bs.00 g421070.m01^p^	2,101	12	−177	−2.63 (.49)	4.44E‐09	Chlorophyll A‐B binding protein
Bs.00 g429620.m01	67	4	−16	−2.63 (.44)	2.53E‐05	Multicopper oxidase
Bs.00 g372170.m02	220	11	−20	−2.62 (.46)	6.13E‐06	Uncharacterized protein

	Flur‐R untreated	Flur‐R 10 HAT				
Bs.00 g253530.m01^u^	3,988	31	−131	−6.56 (.29)	2.06E‐91	Tetratricopeptide‐like helical domain superfamily
Bs.00 g240870.m01^u^	389,614	3,606	−108	−6.40 (.26)	1.25E‐104	Chlorophyll A‐B binding protein
Bs.00 g240870.m02^u^	246,956	2,360	−105	−6.35 (.27)	5.04E‐101	Chlorophyll A‐B binding protein
Bs.00 g060850.m01^u^	59,810	959	−62	−5.34 (.36)	1.12E‐37	Thiamine Thiazole synthase
Bs.00 g126000.m01^†^	66,186	447	−148	−5.12 (.50)	4.64E‐19	NADH‐cytochrome B5 reductase
Bs.00 g258890.m01^†^	136	1	−126	−5.10 (.48)	1.28E‐14	Tyrosine‐protein kinase, active site
Bs.00 g205780.m01	1,166	26	−44	−4.99 (.32)	8.66E‐41	Cytochrome P450 90A1
Bs.00 g133350.m02	459	11	−44	−4.88 (.34)	8.43E‐35	Serine protease family S10 serine carboxypeptidase
Bs.00 g104620.m01^e^ ^†^	7,356	100	−73	−4.81 (.45)	7.39E‐21	Phosphoenolpyruvate carboxylase kinase 1‐related
Bs.00 g133350.m01	507	12	−41	−4.69 (.36)	3.06E‐28	Peptidase S10, serine carboxypeptidase
Bs.00 g192330.m01	293,420	9,287	−32	−4.60 (.31)	1.24E‐37	Magnesium‐chelatase, subunit H
Bs.00 g279710.m01^f^	7,847	165	−48	−4.59 (.42)	6.82E‐20	Aerolysin‐like toxin
Bs.00 g299020.m01	794	16	−50	−4.58 (.44)	9.29E‐19	O‐acyltransferase WSD1
Bs.00 g116620.m01	2,862	91	−31	−4.54 (.31)	2.96E‐37	Coenzyme Q‐binding protein Coq10
Bs.00 g480400.m01^v^	498	8	−62	−4.52 (.49)	4.12E‐14	Plc‐like phosphodiesterase
Bs.00 g206320.m01	10,980	18	−609	−4.48 (.58)	5.73E‐15	Cytochrome P450 superfamily
Bs.00 g058520.m01^u^	1710	65	−26	−4.47 (.26)	1.29E‐47	Acyl‐CoA N‐acyltransferases
Bs.00 g205800.m01	868	23	−38	−4.45 (.41)	1.42E‐19	Cytochrome P450 90A1
Bs.00 g403960.m01^†^	36,087	952	−38	−4.45 (.38)	4.49E‐23	Carotenoid cleavage dioxygenase 4, Chloroplastic‐related
Bs.00 g471400.m01	279	6	−43	−4.44 (.43)	3.69E‐17	Voltage‐gated potassium channels

^b^ Shared between J01‐S 3 HAT and Flur‐R 3 HAT top 20 downregulated genes.

^e^ Shared between J01‐S 3 HAT and Flur‐R 3 HAT/10 HAT top 20 downregulated genes.

^f^ Shared between J01‐S 10 HAT and Flur‐R 10 HAT top 20 downregulated genes.

^p^ Shared between 9,425‐S 3 HAT, J01‐S 3 HAT, and Flur‐R 3 HAT top 20 downregulated genes.

^r^ Shared between 9,425‐S 3 HAT and Flur‐R 3 HAT top 20 downregulated genes.

^v^ Shared between 9,425‐S 10 HAT and Flur‐R 10 HAT top 20 downregulated genes.

^†^ Shared between Flur‐R 3 HAT/10 HAT top 20 downregulated genes.

**TABLE 7:** Top 20 downregulated genes in fluroxypyr susceptible kochia ( *Bassia scoparia* ) line J01‐S at 3 h after treatment (HAT) and 10 HAT compared to the untreated timepoint. fold change was calculated using the mean of normalized counts which was produced using the DESeq2 package in R. Log2 fold change was calculated in DESeq2, log2 fold change standard error and adjusted *p*‐value were also calculated in DESeq2. The Wald‐test obtained *p*‐values were adjusted using the Benjamini‐Hochberg method. The FDR was <.05.

Gene ID	Mean of normalized counts		Fold change	Log2 fold change ( ±SE)	*p*‐value	Gene description
	J01‐S untreated	J01‐S 3 HAT				
Bs.00 g420960.m01^p^	2,805	114	−25	−7.08 (.43)	3.21E‐37	Early light‐induced protein 1, Chloroplastic‐related
Bs.00 g421070.m01^p^	3,659	129	−28	−6.40 (.46)	1.24E‐31	Early light‐induced protein 1, Chloroplastic‐related
Bs.00 g104620.m01^e †^	6,485	87	−74	−5.07 (.37)	1.81E‐33	Phosphoenolpyruvate carboxylase kinase 1‐related
Bs.00 g518390.m01^o^	1,330	91	−15	−4.38 (.45)	8.59E‐17	Chalcone/stilbene synthase
Bs.00 g383340.m01^q^ ^†^	62,541	614	−102	−4.23 (.38)	5.29E‐22	S‐Adenosyl‐L‐methionine‐dependent methyltransferase
Bs.00 g383340.m02^q^ ^†^	63,412	627	−101	−4.22 (.38)	6.99E‐22	S‐Adenosyl‐L‐methionine‐dependent methyltransferase
Bs.00 g479050.m01	1,337	71	−19	−4.00 (.37)	2.08E‐19	Multicopper oxidase, type 1, 2, 3
Bs.00 g150590.m01	19,150	874	−22	−3.52 (.23)	6.20E‐37	Carboxyvinyl‐Carboxyphosphonate Phosphorylmutase, Chloroplastic
Bs.00 g417520.m01	3,342	727	−5	−3.51 (.46)	1.48E‐09	Cytochrome P450 86a7
Bs.00 g196880.m01	12,374	621	−20	−3.40 (.29)	7.21E‐22	Haloacid dehalogenase‐like hydrolase domain‐containing protein
Bs.00 g228740.m01	3,131	464	−7	−3.36 (.43)	4.71E‐10	Glucose‐methanol‐choline oxidoreductase
Bs.00 g488230.m02^s^	493	30	−16	−3.27 (.39)	4.26E‐11	BTB/POZ domain‐containing protein Dot3
Bs.00 g091650.m01	5,414	1,641	−3	−3.24 (.40)	1.94E‐10	Cytochrome P450 77A4‐related
Bs.00 g179870.m01	560	21	−27	−3.20 (.48)	1.00E‐07	Thioredoxin‐LIK
Bs.00 g488230.m01^s^	589	39	−15	−3.15 (.38)	3.15E‐11	BTB/POZ domain‐containing protein DOT3
Bs.00 g124100.m01	25,716	1786	−14	−3.14 (.37)	1.34E‐11	Multi antimicrobial extrusion protein
Bs.00 g195790.m01^b^	661	34	−20	−3.11 (.53)	1.62E‐06	Proton‐dependent oligopeptide transporter family
Bs.00 g247060.m01	1,115	125	−9	−3.08 (.36)	3.19E‐11	Major facilitator protein
Bs.00 g418430.m01	2076	95	−22	−3.05 (.24)	3.85E‐24	Peptidase S10, serine carboxypeptidase
Bs.00 g310680.m01	4,294	815	−5	3.02 (.39)	3.14E‐09	Aquaporin transporter

	J01‐S untreated	J01‐S 10 HAT				
Bs.00 g237950.m01	31,050	185	−168	−6.11 (.47)	2.24E‐29	Purine and uridine phosphorylases
Bs.00 g383340.m01^q^ ^†^	62,541	614	−102	−5.98 (.34)	5.59E‐55	S‐Adenosyl‐L‐methionine‐dependent methyltransferase
Bs.00 g383340.m02^q^ ^†^	63,412	627	−101	−5.97 (.34)	7.93E‐55	S‐Adenosyl‐L‐methionine‐dependent methyltransferase
Bs.00 g060850.m01^u^	61,097	963	−63	−5.71 (.25)	7.00E‐96	Thiamine Thiazole synthase
Bs.00 g253530.m01^u^	3,380	51	−66	−5.61 (.30)	2.91E‐59	Tetratricopeptide‐like helical domain superfamily
Bs.00 g104620.m01^e^ ^†^	6,485	87	−74	−5.57 (.33)	7.03E‐51	Phosphoenolpyruvate carboxylase kinase 1‐related
Bs.00 g002550.m01^t^	6,898	56	−123	−5.53 (.50)	1.28E‐19	Glucose‐6‐phosphate/phosphate translocator 2, Chloroplastic
Bs.00 g058520.m01^u^	2,423	35	−69	−5.51 (.36)	1.50E‐39	Acyl‐CoA N‐acyltransferase
Bs.00 g279710.m01^f^	25,350	388	−65	−5.38 (.41)	1.01E‐26	Aerolysin‐like toxin
Bs.00 g240870.m01^u^	407,872	5,349	−76	−5.26 (.45)	3.48E‐22	Chlorophyll A‐B binding protein
Bs.00 g240870.m02^u^	265,402	3,480	−76	−5.24 (.46)	1.70E‐21	Chlorophyll A‐B binding protein
Bs.00 g236450.m01	13,388	387	−35	−4.88 (.24)	4.84E‐72	Protein proton gradient regulation 5
Bs.00 g115330.m01	71,272	1829	−39	−4.85 (.34)	7.75E‐34	Photosystem I reaction center subunit iv A, Chloroplastic‐related
Bs.00 g020870.m01	209,812	6,578	−32	−4.69 (.28)	3.26E‐46	Chlorophyll A/B binding protein
Bs.00 g412900.m01	18,823	572	−33	−4.65 (.31)	1.54E‐39	Granule‐bound starch synthase 1, Chloroplastic/amyloplastic
Bs.00 g476760.m01	699	11	−62	−4.63 (.50)	4.10E‐14	Z − 3‐Hexen‐1‐Ol acetyltransferase
Bs.00 g527540.m01	7,607	68	−112	−4.47 (.58)	7.97E‐10	Proteinase inhibitor I3, Kunitz legume
Bs.00 g472680.m01	1,623	58	−28	−4.35 (.36)	3.49E‐23	Uncharacterized protein
Bs.00 g383860.m01	33,142	1,574	−21	−4.31 (.15)	7.02E‐130	Fructose‐1,6‐Bisphosphatase‐related
Bs.00 g055990.m01	635	21	−30	−4.28 (.40)	3.67E‐19	Uncharacterized protein

^b^ Shared between J01‐S 3 HAT and Flur‐R 3 HAT top 20 downregulated genes.

^e^ Shared between 9,425‐S 3 HAT, J01‐S 3 HAT/10 HAT and Flur‐R 3 HAT/10 HAT top 20 downregulated genes.

^f^ Shared between J01‐S 10 HAT and Flur‐R 10 HAT top 20 downregulated genes.

^o^ Shared between 9,425‐S 3 HAT/10 HAT and J01‐S 3 HAT top 20 downregulated genes.

^p^ Shared between 9,425‐S 3 HAT, J01‐S 3 HAT, and Flur‐R 3 HAT top 20 downregulated genes.

^q^ Shared between 9,425‐S 3 HAT and J01‐S 3 HAT/10 HAT top 20 downregulated genes.

^s^ Shared between 9,425‐S 3 HAT and J01‐S 3 HAT top 20 downregulated genes.

^t^ Shared between 9,425‐S 10 HAT and J01‐S 10 HAT top 20 downregulated genes.

^u^ Shared between 9,425‐S 10 HAT, J01–10 HAT, Flur‐R 10 HAT top 20 downregulated genes.

^w^ Shared between 9,425‐S 10 HAT and J01‐S 3 HAT top 20 downregulated genes.

**TABLE 8:** Top 20 downregulated genes in fluroxypyr susceptible kochia ( *Bassia scoparia* ) line 9,425 at 3 hours after treatment (HAT) and 10HAT compared to the untreated timepoint. fold change was calculated using the mean of normalized counts which was produced using the DESeq2 package in R. Log2 fold change was calculated in DESeq2, log2 fold change standard error and adjusted *p*‐value were also calculated in DESeq2. The Wald‐test obtained *p*‐values were adjusted using the Benjamini‐Hochberg method. The FDR was <.05.

Gene ID	Mean of normalized counts		Fold change	Log2 fold change ( ±SE)	Pvalue	Gene description
	9,425 untreated	9,425 3 HAT				
Bs.00 g518390.m01^o^ ^†^	4,504	68	67	−5.06	1.80E‐41	Chalcone/stilbene synthase
Bs.00 g421070.m01^p^	1918	8	238	−3.69	1.06E‐14	Early light‐induced protein 1, Chloroplastic‐related
Bs.00 g420960.m01^p^	1,557	7	210	−3.61	9.55E‐14	Early light‐induced protein 1, Chloroplastic‐related
Bs.00 g543360.m01	2,375	98	24	−3.38	1.15E‐11	Oxoglutarate/iron‐dependent dioxygenase
Bs.00 g383340.m01^q^	13,230	691	19	−3.31	1.70E‐12	S‐Adenosyl‐L‐methionine‐dependent methyltransferase
Bs.00 g383340.m02^q^	13,404	701	19	−3.31	1.82E‐12	S‐Adenosyl‐L‐methionine‐dependent methyltransferase
Bs.00 g354480.m01^r^	1,542	87	18	−3.25	1.69E‐11	S‐Adenosyl‐L‐methionine‐dependent methyltransferase
Bs.00 g370120.m01^r^	364	8	45	−3.20	6.65E‐09	CRAL‐TRIO lipid binding domain
Bs.00 g268300.m01^†^	724	5	140	−3.16	2.50E‐10	Early light‐induced protein 1, Chloroplastic‐related
Bs.00 g364920.m01	453	37	12	−3.13	1.87E‐15	Serine–threonine/tyrosine‐protein kinase, catalytic domain
Bs.00 g488230.m01^s^	532	38	14	−3.11	2.63E‐12	BTB/POZ domain‐containing protein DOT3
Bs.00 g488230.m02^s^	447	37	12	−3.02	1.55E‐12	BTB/POZ domain‐containing protein DOT3
Bs.00 g475340.m01	478	38	13	−2.95	1.42E‐09	Camp‐response element binding protein‐related
Bs.00 g528960.m01^r^	880	51	17	−2.89	1.17E‐07	Auxin_Inducible
Bs.00 g180610.m01	1,127	60	19	−2.86	1.87E‐07	Glycoside hydrolase family 1
Bs.00 g251440.m01^r^	1,535	140	11	−2.81	4.78E‐10	Multicopper oxidase, type 2
Bs.00 g396960.m01	646	57	11	−2.70	3.62E‐08	UDP‐glycosyltransferase/glycogen phosphorylase
Bs.00 g420270.m01	1,575	133	12	−2.67	2.39E‐06	Glucose‐methanol‐choline oxidoreductase
Bs.00 g104620.m01^e^	1,497	106	14	−2.67	9.72E‐07	Serine/threonine‐protein kinase
Bs.00 g264170.m01^r^	11,365	1,301	9	−2.66	1.13E‐10	Beta‐glucosidase 1‐related

	9,425‐S untreated	9,425 10 HAT				
Bs.00 g002550.m01^t^	10,014	41	242	−6.95	3.36E‐47	Glucose‐6‐phosphate/phosphate translocator 2, Chloroplastic
Bs.00 g518390.m01^o^ ^†^	4,504	21	213	−6.94	7.61E‐75	Chalcone/stilbene synthase, C‐terminal
Bs.00 g240870.m01^u^	419,016	3,501	120	−6.09	2.32E‐35	Chlorophyll A‐B binding protein
Bs.00 g240870.m02^u^	274,431	2,301	119	−6.09	4.32E‐35	Chlorophyll A‐B binding protein
Bs.00 g058520.m01^u^	3,828	37	103	−6.08	4.85E‐49	Acyl‐CoA N‐acyltransferase
Bs.00 g060850.m01^u^	105,278	1,179	89	−5.75	2.70E‐36	Thiamine Thiazole synthase
Bs.00 g253530.m01^u^	3,725	49	76	−5.63	1.99E‐37	Tetratricopeptide‐like helical domain superfamily
Bs.00 g535160.m01	480	4	110	−5.57	2.65E‐21	WAT1‐related protein
Bs.00 g124870.m01	18,471	116	160	−5.33	8.61E‐16	Lipoxygenase
Bs.00 g480400.m01^v^	684	8	85	−5.33	1.31E‐20	PLC‐like phosphodiesterase
Bs.00 g418430.m01	3,350	46	73	−5.33	6.52E‐27	Peptidase S10, serine carboxypeptidase
Bs.00 g535430.m01	143	1	108	−5.30	7.70E‐15	Cyclin_A_B_D_E
Bs.00 g518030.m01	721	2	412	−5.13	1.43E‐12	Chalcone/stilbene synthase, polyketide synthase, type III, Hydroxymethylglutaryl‐CoA synthase
Bs.00 g055200.m01	754	15	51	−5.06	7.48E‐28	Glyceraldehyde‐3‐phosphate dehydrogenase‐like
Bs.00 g535430.m02	121	1	92	−5.05	2.97E‐13	Cyclin_A_B_D_E
Bs.00 g249240.m01	212	2	117	−5.02	5.87E‐13	Uncharacterized protein
Bs.00 g268300.m01^†^	724	4	171	−5.01	6.54E‐14	Early light‐induced protein 1
Bs.00 g435140.m01	1,573	29	55	−4.95	4.56E‐22	Proton‐dependent oligopeptide transporter family
Bs.00 g136920.m01	1931	16	123	−4.92	1.48E‐13	Cupin_1
Bs.00 g478150.m01	781	6	138	−4.90	1.45E‐11	Glycoside hydrolase family 17

^e^ Shared between 9,425 3 HAT, J01–3 HAT/10 HAT and Flur‐R 3 HAT/10 HAT top 20 downregulated genes.

^o^ Shared between 9,425‐S 3 HAT/10 HAT and J01‐S 3 HAT top 20 downregulated genes.

^p^ Shared between 9,425‐S 3 HAT, J01‐S 3 HAT, and Flur‐R 3 HAT top 20 downregulated genes.

^q^ Shared between 9,425‐S 3 HAT and J01‐S 3 HAT/10 HAT top 20 downregulated genes.

^r^ Shared between 9,425‐S 3 HAT and Flur‐R 3 HAT top 20 downregulated genes.

^s^ Shared between 9,425‐S 3 HAT and J01‐S 3 HAT top 20 downregulated genes.

^t^ Shared between 9,425‐S 10 HAT and J01‐S 10 HAT top 20 downregulated genes.

^v^ Shared between 9,425‐S 10 HAT and Flur‐R 10 HAT top 20 downregulated genes.

^w^ Shared between 9,425‐S 10 HAT and J01‐S 3 HAT top 20 downregulated genes.

^†^ Shared between 9,425‐S 3 HAT and 10 HAT top 20 downregulated genes.

### 3.6 Variant analysis

Our decision criteria to determine resistance‐conferring sequence variant candidates were that all four Flur‐R individuals from the RNA‐seq experiment must have the variant. The candidate variant also must be absent in the two S lines (9,425 and J01‐S). Of the 147 genes annotated as CYP450s in the kochia genome, there were no unique variants in the Flur‐R line. There were 37 genes annotated as having an Aux/IAA domain or function and 21 genes with an ARF domain or function. Of these genes, three genes contained a nonsynonymous mutation or a deletion. ARF19/7 (*Bs.00 g057730.m01*), one of five transcriptional activators in the ARF family, had two nonsynonymous mutations (Gly446Ser; Leu486Ile) and two single codon deletions (Figure S3A). A protein annotated as ARF3 (*Bs.00 g076170.m01*) also known as ETTIN (ETT) showed one nonsynonymous homozygous variant (Leu293Ser), where three J01‐S individuals were heterozygous for the variant found in Flur‐R, one was homozygous, and the remaining four 9,425 were wildtype. *Aux/IAA4‐like* (*Bs.00 g107340.m01*) displayed one nonsynonymous variant (Glu52Arg) in a non‐conserved region 6–10 bases N terminal of the Aux/IAA Domain II described by Ramos et al. (2001) (Figure S3B). We also determined there were no variants in any proteins annotated as AFB or TIR1 proteins that were unique to Flur‐R and met our specified criteria, and there were no mutations in the 18 LRR‐rich C terminus where Aux/IAA and auxin are reported to bind (Villalobos et al., 2012).

## 4 DISCUSSION AND CONCLUSION

Flur‐R, 9425, and J01‐S all had up‐regulation of auxin‐regulated genes in response to fluroxypyr that was similar to the auxin mimic herbicide gene expression response in Arabidopsis (Gleason et al., 2011; Goda et al., 2004). The increased expression of auxin‐responsive genes following fluroxypyr treatment suggests that fluroxypyr is being perceived similarly by all three lines and supports our findings that target‐site variants found in *Aux/IAA4* and *ARF19/7* are likely not the cause of the fluroxypyr resistance response in Flur‐R. Specifically in *ARF19/7*, the identified variants are predicted to have no significant effect on fluroxypyr binding due to their position in the variable middle region described by Ulmasov et al. (1999) (Figure S3A). Although we did find a Flur‐R homozygous variant in *ARF3*, the region boundaries of *ARF3* are unlike most other ARFs in that it does not contain Domain III/IV, two key domains for interaction with Aux/IAA proteins relating to auxin gene expression. ARF3 does function in some auxin‐related pathways (reviewed by Liu et al., 2014) but the protein has been described to function as a repressor of several proteins causing inhibition of cytokinin activity, a plant hormone that often partners with IAA (Zhang et al., 2018). While we cannot be certain that the variant in *ARF3* does not contribute indirectly to fluroxypyr resistance or affect cytokinin levels in the plant, due to the ARF3 described function, there is stronger support for metabolism being the underlying cause of resistance. Additionally, if target‐site variants were present that affected auxin‐mimic perception or binding, such as the *IAA16* Gly127Asn mutation described by LeClere et al. (2018), the expected auxin‐response gene expression would likely not be induced as reported by Pettinga et al. (2018) in the 9,425 line when tested with the auxin‐mimic herbicide dicamba.

The translocation data suggest that fluroxypyr, being primarily in its acid form based on the 6 h metabolism results, is moving symplastically throughout the plant as a phloem mobile herbicide (Schober et al., 1986). Transcripts for two IAA transporter (PIN) isoforms were upregulated in the susceptible lines 9,425 and J01‐S when treated with fluroxypyr, suggesting that PINs can transport fluroxypyr in a similar manner to the transport of IAA. Based on the lack of differences in translocation between Flur‐R and J01‐S, these two identified PIN transporters are not moving phytotoxic fluroxypyr acid throughout the resistant or susceptible plants at a different rate. Other transporters such as ATP binding cassettes (ABCs) in class B can move multiple substrates including xenobiotics. Some members of this large protein family serve as auxin transporters (Cho & Cho, 2013). ABC transporters from both class B and G were upregulated in Flur‐R following fluroxypyr treatment, none of which have been individually implicated in herbicide resistance. Several class G transporters are involved in auxin homeostasis and other phytohormone transport, cellular detoxification of heavy metals, and pathogen resistance (Dhara & Raichaudhuri, 2021; Gräfe & Schmitt, 2021). The functional suite of ABC class G transporters in kochia is yet to be fully described, though cellular export of fluroxypyr conjugates is not outside the scope of known activity for class G transporters.

In Flur‐R, abscisic acid (ABA) biosynthesis gene *NCED2* transcript expression decreased over a 10 h time period, contrasting the results from the two susceptible lines in which *NCED6* transcripts had increased expression at 3 h in all three lines (Figure 8). The implications of decreased *NCED2* expression in the resistant line are currently unknown, though some reports show an increased level of response from *NCED* genes following various auxin‐mimic applications (Kraft et al., 2007; McCauley et al., 2020; Raghavan et al., 2005). Among these ABA‐related downregulated genes, seven subunits of Photosystem I and four subunits of Photosystem II are downregulated in all three lines following fluroxypyr application suggesting that fluroxypyr may affect light energy harvesting as part of its mechanism of action. These findings are consistent with the findings of McCauley et al. (2020).

Of the five CYP450s constitutively expressed in Flur‐R compared to either 9,425 or J01‐S, *CYP71D10/11* has been implicated in metabolic herbicide resistance to fenoxaprop‐p‐ethyl (Bai et al., 2020). Other CYP450s in the CYP71 family have been described as shikimate and shikimate intermediate modifiers (Jun et al., 2015), including Ent‐kaurene oxidase (CYP701 subfamily) which functions in gibberellin biosynthesis; its overexpression causes partial resistance to plant growth retardant uniconazole‐P (Miyazaki et al., 2011). *CYP81B2* (*Bs.00 g431990.m01*) in transgenic tobacco metabolized the phenylurea herbicide chlortoluron after the application of auxin‐mimic 2,4‐D. The same study also identified its involvement in secondary metabolite biosynthesis (Ohkawa et al., 1999). The other two treatment‐induced CYP450s in Flur‐R, *CYP82D47*, and *CYP71A9‐like*, have no described role in herbicide resistance, however, there are a significant number of CYP450s involved in herbicide metabolism in the CYP71 family, to which they both belong (Gion et al., 2014; Siminszky et al., 1999; Xiang et al., 2006).

The final constitutively expressed CYP450 in the Flur‐R line, *CYP90C1/D1*, belongs to the CYP85 family which is implicated in modification of cyclic terpenes and sterols in brassinosteroid, abscisic acid, and gibberellin biosynthesis (Jun et al., 2015; Ohnishi et al., 2006; Ohnishi et al., 2012). It is not unusual for CYP450s to be multifunctional (Bernhardt, 2006), and their function can often be attributed to the selectivity of some herbicides, extensively reviewed by Dimaano and Iwakami (2021).

We investigated fluroxypyr resistance using herbicide physiology experiments as well as RNA‐sequencing and identified metabolic detoxification as a plausible explanation of fluroxypyr resistance in Flur‐R. Two of the four metabolites are accounted for, having been reported by the Environmental Protection Agency (EPA, 2010). The action of conjugation by GSTs or GTs may explain one of the two remaining undescribed metabolites presented, which were both rapidly converted from fluroxypyr acid throughout the time course in Flur‐R. Given the high expression of five GSTs and eight GTs in both untreated and treatment‐induced conditions, the formation of secondary metabolic structures is possible. Following CYP450 activity via *O*‐glucosylation, fluroxypyr‐tripeptide GST or ‐sugar conjugates can be catalyzed by GST or UDP‐glucosyl transferase (Ludwig‐Müller, 2011). GSTs and GTs can glycosylate plant hormones and xenobiotics to influence bioactivity, transport, and solubility and can be pumped out of the cell via ABC transporters (Li et al., 2001; Moons, 2005). Subsequent sequestration of the non‐phytotoxic herbicide via ABC transporter may also play a role in the resistance response in Flur‐R, though more work is needed to fully understand the metabolic response following fluroxypyr application in Flur‐R.

In conclusion, Flur‐R converted fluroxypyr‐ester into fluroxypyr‐acid and subsequent metabolites at a faster rate than a susceptible line. Furthermore, the resistant line produced a metabolite that was not detected in the susceptible line. The results from an RNA‐seq fluroxypyr‐induced differential expression analysis showed increased transcript expression of cellular transporters, cytochrome P450 monooxygenases (CYP450), glutathione s‐transferases (GSTs), and UDP‐glucosyl transferase (GTs) in the resistant plants. Taken together, these data suggest that metabolic detoxification of fluroxypyr may be the mechanism of fluroxypyr resistance in Flur‐R.

## 5 FUTURE WORK

Future work elucidating the fluroxypyr resistance mechanism involves in vitro and in vivo testing of the five candidate GSTs, eight GTs, and eight CYP450s. Metabolite identification via LCMS/MS is a crucial next step to determine the metabolic path of the fluroxypyr molecule. Other future studies include genetic mapping of fluroxypyr resistance via test crosses and either Quantitative Trait Loci (QTL) or bulk‐segregant analysis with resistant Flur‐R and susceptible J01‐S, which will provide chromosomal location of resistance gene(s) (Montgomery et al., 2023). Biochemical studies using P450 and GST inhibitors will indicate whether the enhanced fluroxypyr metabolism can be reversed. Metabolic information paired with mapping and ongoing inheritance studies will be a strong contribution to the understanding of auxin‐mimic resistance and the characterization of fluroxypyr resistance in this population. Identifying causal resistance genes in auxin‐mimic resistant kochia populations will allow us to document the evolution of new resistance genes and predict patterns of gene flow, following the model set by Ravet et al. (2021) for gene flow of glyphosate resistance in kochia.

## AUTHOR CONTRIBUTIONS USING CREDIT AUTHOR STATEMENTS

**Olivia E. Todd:** Writing, visualization, data curation, investigation, formal analysis, validation, methodology **Eric L. Patterson:** Resources, methodology **Eric P. Westra:** Resources **André Lucas Simões Araujo:** Investigation **William B. Kramer:** Investigation **Franck E. Dayan:** Methodology, supervision, resources **Scott J. Nissen:** Methodology, resources **Todd A. Gaines:** Writing, supervision, conceptualization.

## CONFLICT OF INTEREST STATEMENT

The Authors did not report any conflict of interest.

## PEER REVIEW

The peer review history for this article is available in the Supporting Information for this article.

## Supporting information


**Table S1.** Summary of RNA‐Seq reads and mapping results. Total read counts, total unmapped reads, uniquely mapped reads, and multimapped reads following BGI‐seq on fluroxypyr resistant kochia population Flur‐R and two fluroxypyr susceptible populations, J01‐S and 9,425. Three treatments in the RNA‐seq study included untreated, “UNT”; 3 hours after treatment with fluroxypyr, “3HAT”; and 10 hours after treatment with fluroxypyr, “10HAT”. Results are taken from the summary text resulting from an alignment of reads to the coding sequence by HISAT2 and feature assignment using featureCounts. Total reads are calculated from the number of pairs. Uniquely mapped reads are calculated from the number of neither concordant nor discordant aligning pairs. Multimapped reads are calculated from the number of concordant pairs that aligned more than once to a given area.

**Figure S1.** Sequence analysis of four Flur‐R individuals from the RNA‐sequencing data show that there is a Proline 197 to Threonine (P197T) mutation and a Tryptophan 574 to Leucine (W574L) mutation in the acetolactate synthase gene. Resistance to ALS herbicides due to these mutations is a dominant or semi‐dominant trait, in which heterozygosity is sufficient to confer resistance to ALS‐inhibiting herbicides.

**Figure S2.** Expression profiles for auxin‐induced genes GH3.2, ACS, and various high annotation confidence Aux/IAAs in fluroxypyr resistant kochia ( *Bassia scoparia* ) Flur‐R, susceptible J01‐S, and susceptible 9,425‐S following differential expression analysis of RNA‐Seq data. X‐axis shows treatments: untreated, 3 h after treatment (HAT), and 10 HAT grouped by kochia line. Normalized counts on the y‐axis are a result of the DESeq2 function and model fitting in the R package “DESeq2”.

**Figure S3.** A. Variants in the gene ARF 19/7 (Bs.00 g057730.m01). Two nonsynonymous mutations (Gly446Ser; Leu486Ile) are represented by white markers, and two single codon deletions are represented by black markers. B. Aux/IAA 4 (Bs.00 g107340.m01) nonsynonymous mutation (Glu52Arg) in the N terminal region of Domain II present in the Flur‐R line.

**Data S1.** Supporting Information.

## Data Availability

The data underlying this article are available in the Gene Expression Omnibus at https://www.ncbi.nlm.nih.gov/geo/query/acc.cgi?acc=GSE179578, and can be accessed with GEO Accession GSE179578.
