# Supplementary figures and images for: Enhanced metabolic detoxification is associated with fluroxypyr resistance in Bassia scoparia
Source: Plant Direct. 2024 Jan 24;8(1):e560. doi: 10.1002/pld3.560 (PMC10807189; doi:10.1002/pld3.560)

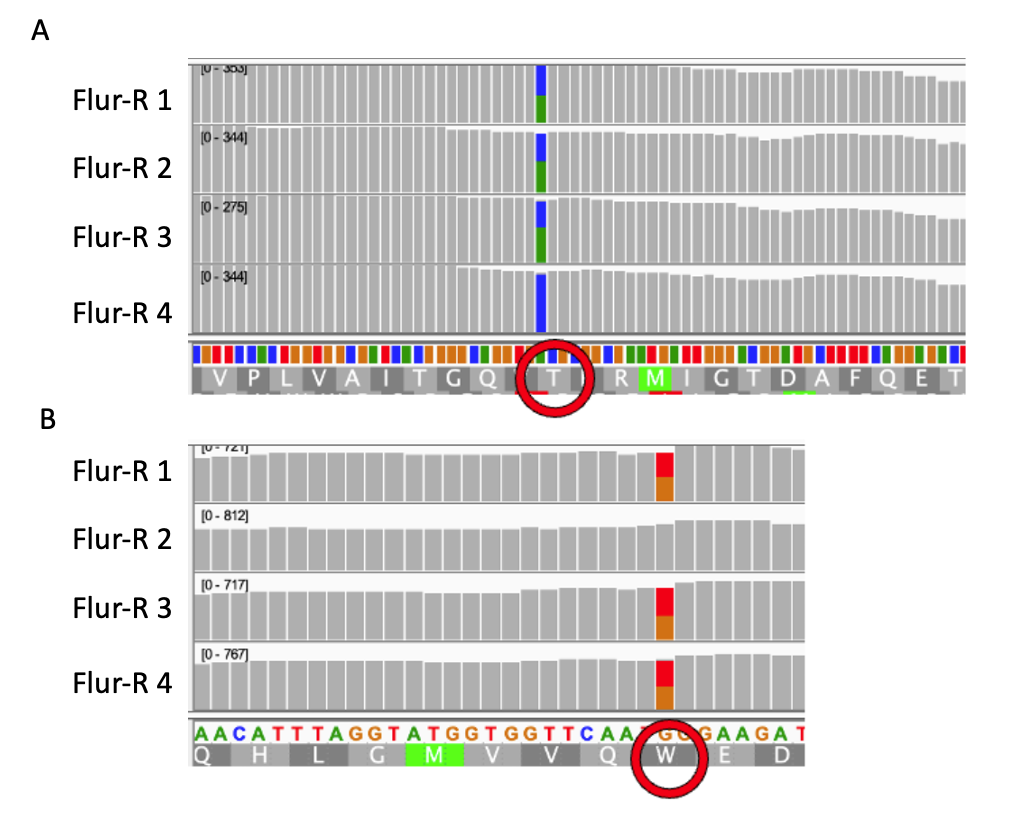

Supplement: Supplementary file 2 — Figure S1. Sequence analysis of four Flur‐R individuals from the RNA‐sequencing data show that there is a Proline 197 to Threonine (P197T) mutation and a Tryptophan 574 to Leucine (W574L) mutation in the acetolactate synthase gene. Resistance to ALS herbicides due to these mutations is a dominant or semi‐dominant trait, in which heterozygosity is sufficient to confer resistance to ALS‐inhibiting herbicides. [file PLD3-8-e560-s001.png]

**Aux/IAA29 (Bs.00g048560.m01)**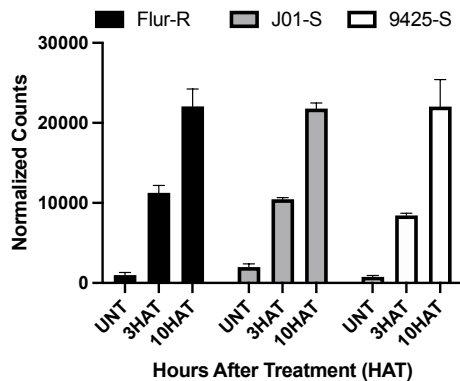**Aux/IAA16 (Bs.00g107550.m01)**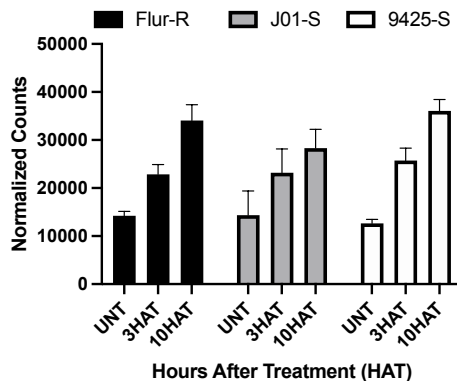**Aux/IAA31 (Bs.00g220790.m01)**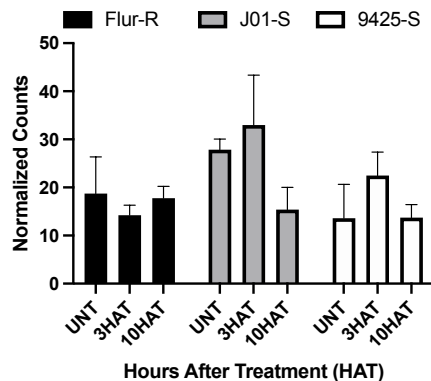**Aux/IAA12 (Bs.00g258500.m01)**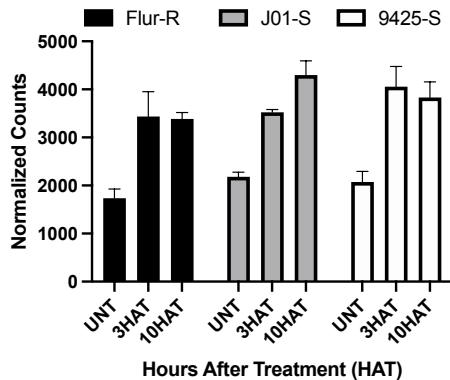**ACS (Bs.00g478760.m01)**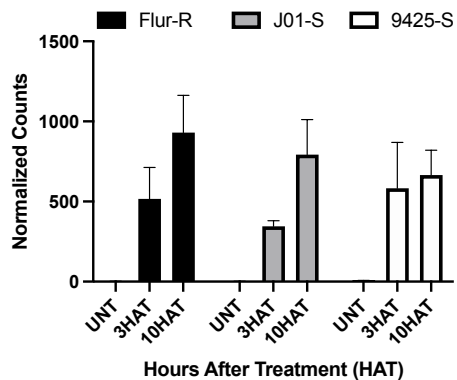**GH3.2-related (Bs.00g477580.m01)**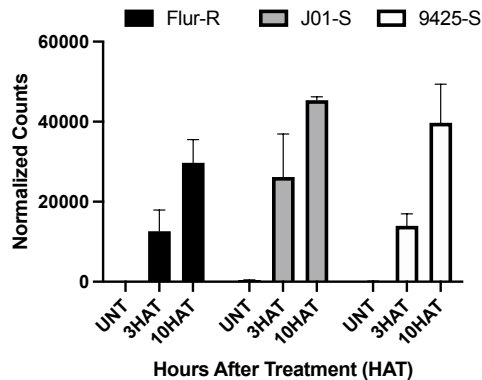

Supplement: Supplementary file 3 — Figure S2. Expression profiles for auxin‐induced genes GH3.2, ACS, and various high annotation confidence Aux/IAAs in fluroxypyr resistant kochia ( Bassia scoparia ) Flur‐R, susceptible J01‐S, and susceptible 9,425‐S following differential expression analysis of RNA‐Seq data. X‐axis shows treatments: untreated, 3 h after treatment (HAT), and 10 HAT grouped by kochia line. Normalized counts on the y‐axis are a result of the DESeq2 function and model fitting in the R package “DESeq2”. [file PLD3-8-e560-s003.pdf]

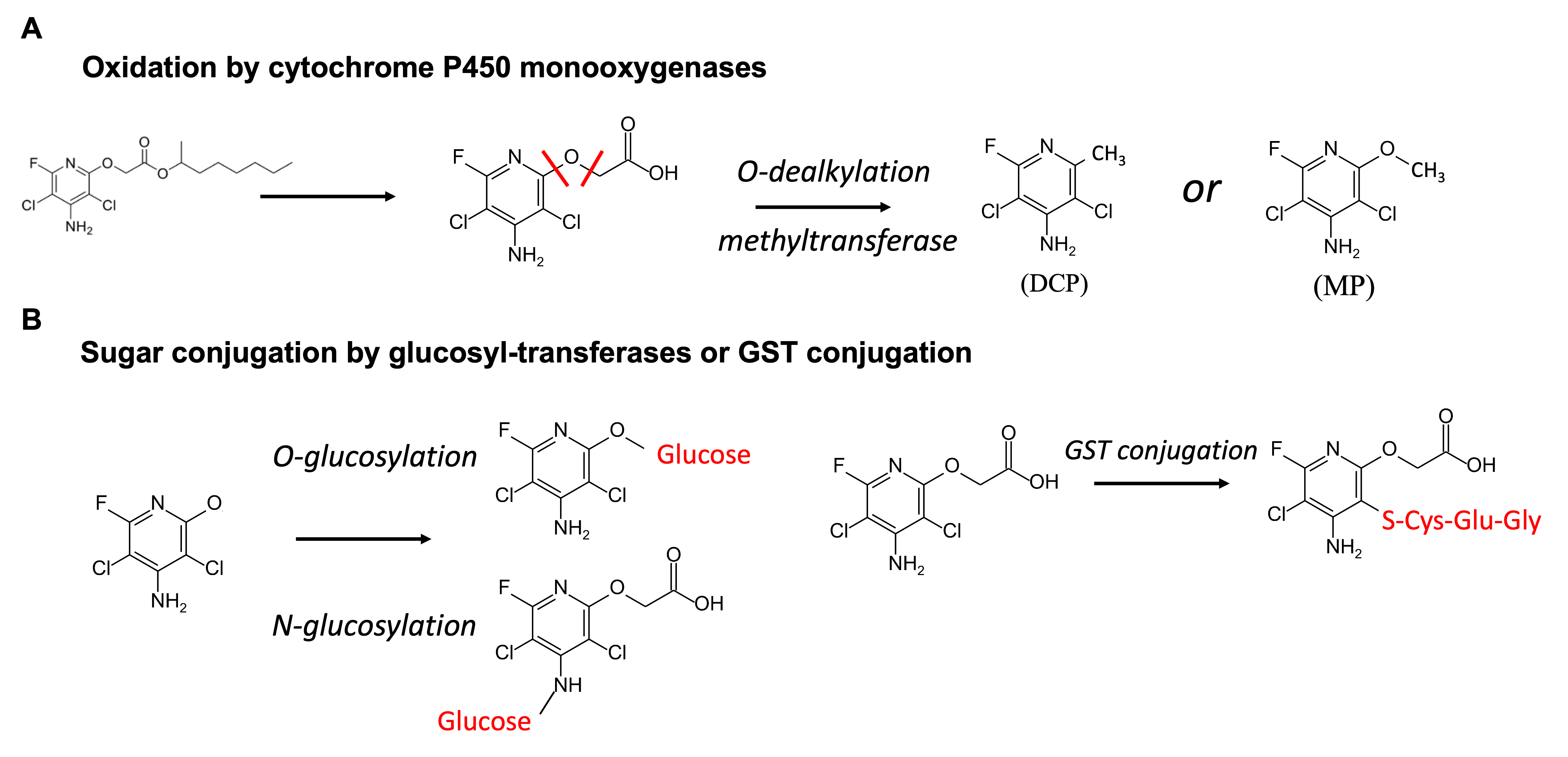

Supplement: Supplementary file 4 — Figure S3. A. Variants in the gene ARF 19/7 (Bs.00 g057730.m01). Two nonsynonymous mutations (Gly446Ser; Leu486Ile) are represented by white markers, and two single codon deletions are represented by black markers. B. Aux/IAA 4 (Bs.00 g107340.m01) nonsynonymous mutation (Glu52Arg) in the N terminal region of Domain II present in the Flur‐R line. [file PLD3-8-e560-s005.png]
