# Supplementary material for: Enhanced metabolic detoxification is associated with fluroxypyr resistance in Bassia scoparia
Source: Plant Direct. 2024 Jan 24;8(1):e560. doi: 10.1002/pld3.560 (PMC10807189; doi:10.1002/pld3.560)
Supplement: Supplementary file 5 — Data S1. Supporting Information. [file PLD3-8-e560-s002.pdf]

Plant Direct
Open Access

Enhanced metabolic detoxification is associated with fluroxypyr resistance in *Bassia scoparia*

Olivia E. Todd[1,2], Eric L. Patterson[3], Eric P. Westra[2,4], Scott J. Nissen[2], André Lucas Simões Araujo[2], William B. Kramer[2], Franck E. Dayan[2], Todd A. Gaines[2]*

DECISION LETTER- ROUND 1

November 27, 2023

Dr. Todd A. Gaines
Colorado State University
Bioagricultural Sciences and Pest Management
1177 Campus Delivery
Fort Collins, Colorado 80523

RE: Enhanced metabolic detoxification is associated with fluroxypyr resistance in Bassia scoparia

Dear Dr. Gaines:

Thank you for submitting to Plant Direct. All required reviews have been returned and we have now finished our evaluation of your manuscript. In light of the reviewers' and editor's comments, further minor revisions are needed before the paper can be accepted for publication in Plant Direct.

Please view the editors' and reviewers' comments below and use their suggestions as a guide while you work on your revision.

When uploading the revised version of this article, please be sure to include the following:

-A word document that contains your response to the reviewers. You should respond to each reviewer comment and note the changes made to the manuscript. If you do not agree with a reviewer's comment and choose not to make a suggested revision, please explain why. Please try to provide as complete an answer as possible to each reviewer's criticisms.
-A tracked changes document with each change highlighted
- A clean version of the latest version of the manuscript

To upload your revision, please click the link below.
https://plantdirect.msubmit.net/cgi-bin/main.plex?el=A6Lr1dI3A6HOQ6I5A9ftdwYnqfX7bO0XNoEqw84ihEQZ

In order to provide as timely a service as possible, we ask that your revision is resubmitted within three months after receipt of this request. If an extension is needed, please send a request, along with a brief explanation, to the editorial office at plantdirect@wiley.com .

Please note that, in addition to publishing reviewer comments, the author's responses to review comments will also be published alongside the final version of the paper. If you would not like the author's responses to be published, please contact the editorial office at plantdirect@wiley.com .

Thank you very much for giving us an opportunity to review your work. I look forward to receiving the next version.

Sincerely,

Marco Giovannetti

Esther van der Knaap

Editor, Plant Direct

-- Reviewer comments:
Reviewer #1:

The manuscript covers an in-depth study documenting enhanced metabolism as the likely mechanism conferring fluroxypyr resistance in kochia from Colorado. Overall, the manuscript is very well written, presented in logical order, and the results support the overall conclusions. Great job on an excellent contribution to the body of knowledge on kochia. Please consider the following suggestions that came to mind when reviewing the manuscript:
L120-121: Add change in leaf angle as one of the phenotypic responses to auxin mimics.
L148-155: This paragraph should either be moved to the conclusions or removed all together. It is redundant with the conclusions of the manuscript and as presented seems out of order.
L170: Supplemental light?
L180: Add 'were' after "plants"
L193-197: What about plants that exhibited only one or two of these symptoms but not the others? How were they categorized?
L202-223: How did you assess for the assumptions of nonlinear regression?
L223: How was the repeat of the experiment dealt with during statistical analysis?
L227: Was it a cell or a pot? You indicate both in this sentence.
L419-423: This is redundant and could be removed.
L438: Remove "from"
L662: Quantitative "Trait" Loci?

Thank you for the opportunity to review this excellent contribution.

Reviewer #2:

Authors are commended for significant amount of work on elucidating the possible mechanism of

fluroxypyr resistance in kochia. Results reported are important and worthy publication to advance our understanding on Auxinic resistance (especially fluroxypyr) in weed species such as kochia. I have provided minor edits for authors to consider on the attached file.

Attachment: Reviewer 2 Review Attachment 1 - 11-27-2023 09:06:24

DECISION LETTER- ROUND 2:

December 6, 2023
Dr. Todd A. Gaines
Colorado State University
Bioagricultural Sciences and Pest Management
1177 Campus Delivery
Fort Collins, Colorado 80523

MSID: 2023-01304R1
MS TITLE: Enhanced metabolic detoxification is associated with fluroxypyr resistance in Bassia scoparia

Dear Dr. Todd Gaines:

I am pleased to inform you that your manuscript "Enhanced metabolic detoxification is associated with fluroxypyr resistance in Bassia scoparia" has been accepted for publication in Plant Direct.

Your article will appear online in the next available issue of Plant Direct. To ensure your article gets published as quickly as possible, please pay attention to the steps detailed below. We have found that most of the delays happen at this stage, especially at the payment stage, so please respond as quickly as possible when prompted.

License Agreement: Once your article has been accepted it will move to Production and undergo admin and file checking - you may receive an email with any queries we have at this stage. When all required items are received by the publisher and queries resolved, the corresponding author will receive an email from Wiley's Author Services system which will ask them to log on at https://authorservices.wiley.com/bauthor and will present them with the appropriate license for completion. Your article cannot be published until both the signed license agreement and payment of the article fee have been received.

Payment of the Open Access Article Publication Fee: All articles published in Plant Direct are fully open access: immediately and freely available to read, download and share. Plant Direct charges a publication fee to cover the publication costs. The corresponding author for this manuscript should have already received a quote from the payments team (cs-openaccess@wiley.com) with the estimated article publication fee; please email cs-openaccess@wiley.com if this has not been received. The corresponding author should log on to the Wiley Author Services site, where the publication fee can be paid by credit card or an invoice. Pro Forma can also be requested. Payment of the publication charge must be received before the article will be published online.

Proofs: You will have the opportunity to look over your paper once more when you receive the

author proofs for your article. The proofs will be with you in approximately two weeks. Please note that, in addition to publishing reviewer comments, the author's responses to review comments will also be published alongside the final version of the paper. If you would not like the author's responses to be published, please contact the editorial office at plantdirect@wiley.com .

Promotion of your article: You can help your research get the attention it deserves! Check out Wiley's free Promotion Guide for best-practice recommendations for promoting your work at www.wileyauthors.com/eeo/guide. And learn more about Wiley Editing Services which offers professional video, design, and writing services to create shareable video abstracts, infographics, conference posters, lay summaries, and research news stories for your research at www.wileyauthors.com/eeo/promotion.

Thank you again for your contribution to Plant Direct. If you have any questions, feel free to contact the editorial office at plantdirect@wiley.com .

Sincerely,

Marco Giovannetti

Esther van der Knaap

Editor, Plant Direct

AUTHORS' RESPONSE

30 November 2023

Plant Direct: 2023-01304

Title: Enhanced metabolic detoxification is associated with fluroxypyr resistance in Bassia scoparia

Revision 1

We thank the reviewers and editor for their assessments of our manuscript and comments to improve the clarity of presentation. We provide our point by point responses to the reviewer comments below. We are submitting a version of the manuscript with Track Changes shown, and a clean version with all Track Changes accepted. We have made minor formatting changes to Figures 4 and 8 (removing headers from graphs and moving the information to the figure legend).

Reviewer #1:

The manuscript covers an in-depth study documenting enhanced metabolism as the likely mechanism conferring fluroxypyr resistance in kochia from Colorado. Overall, the manuscript is very well written, presented in logical order, and the results support the overall conclusions. Great job on an excellent contribution to the body of knowledge on kochia. Please consider the following suggestions that came to mind when reviewing the manuscript:

We thank the reviewer for their time reading through this manuscript, and for their comments to improve it.

L120-121: Add change in leaf angle as one of the phenotypic responses to auxin mimics.

The addition was made.

L148-155: This paragraph should either be moved to the conclusions or removed all together. It is redundant with the conclusions of the manuscript and as presented seems out of order.

We consider this summary of the conclusions to be important to keep. We moved it to the end of the conclusions section as suggested.

L170: Supplemental light?

Yes, with approximately 13 hours of daylight in the spring in Colorado, 3 hours of supplemental light was needed when cloudy or in the evening. We inserted the information about supplemental lighting.

L180: Add 'were' after "plants

The addition was made.

L193-197: What about plants that exhibited only one or two of these symptoms but not the others? How were they categorized?

When fluroxypyr is applied, these symptoms are a well-described suite that often occur together. We saw uniform responses to chemical application, therefore there were no individuals in this experiment that showed an intermediate phenotype. We added this sentence:

"All individuals were uniform for either dead or alive phenotypes, with no intermediate responses."

L202-223: How did you assess for the assumptions of nonlinear regression?

We assessed the assumptions of normality for this dataset: The observations were independent; the homoscedastic property and normality of the data were tested. The model fit was tested using the "ModelFit" from the drc package in R (we inserted this information at lines 224-225).

We added this sentence to the paragraph:

"The homoscedasticity and normality of the data were assessed and met model assumptions."

L223: How was the repeat of the experiment dealt with during statistical analysis?

A homogeneity of variance test was conducted to see if the data could be combined for the repeat of the dose response. The data could not be combined, so the repeated experiment was analyzed separately with repeated results.

We added this sentence to the end of the paragraph:

"A homogeneity of variance test was conducted to determine if the repeated dose response data could be combined for analysis. The data could not be combined, so the repeated experiment was analyzed separately and consistent results were identified."

L227: Was it a cell or a pot? You indicate both in this sentence.

We have clarified this to consistently use the term pot.

L419-423: This is redundant and could be removed.

We made this change.

L438: Remove "from"

We made this change.

L662: Quantitative "Trait" Loci?

We made this change.

Reviewer #2:

Authors are commended for significant amount of work on elucidating the possible mechanism of fluroxypyr resistance in kochia. Results reported are important and worthy publication to advance our understanding on Auxinic resistance (especially fluroxypyr) in weed species such as kochia. I have provided minor edits for authors to consider on the attached file.

We thank the reviewer for their time and effort given to the text suggestions. We made all suggested changes except one, and request to keep the text at this one location as originally stated:

At lines 381-383 in previous version (currently lines 423-425) referring to previous results showing fluroxypyr resistance lin kochia line 9425: We prefer to keep this statement in the text at this point, as we need to carefully point out for the reader that we found a different result than the previous work.
